# Engineered Protein Modification: A New Paradigm for Enhancing Biosensing Sensitivity and Diagnostic Accuracy

**DOI:** 10.3390/bios16010021

**Published:** 2025-12-26

**Authors:** Zheng Xu, Chu Wang, Ziting Zhang, Heng Wang, Peiyi Gao, Lixing Weng

**Affiliations:** State Key Laboratory of Flexible Electronics (LoFE) and Jiangsu Key Laboratory of Smart Biomaterials and Theranostic Technology, Institute of Advanced Materials (IAM), Jiangsu National Synergetic Innovation Center for Advanced Materials (SICAM), Nanjing University of Posts and Telecommunications, Nanjing 210023, China

**Keywords:** protein modifications, biosensing, post-translational modifications, disease diagnosis, food safety, environmental monitoring

## Abstract

Protein modifications, particularly post-translational modifications (PTMs) such as phosphorylation and glycosylation, are fundamental mechanisms regulating cellular activity and disease pathogenesis, with their detection emerging as a promising frontier for advanced diagnostics. This review systematically examines the integration of engineered protein modifications with biosensing technologies to enhance analytical performance and diagnostic accuracy. Through critical analysis of current methodologies, we highlight how strategic manipulation of PTMs improves biosensor sensitivity and specificity in applications ranging from early disease detection to environmental monitoring. The analysis identifies significant advancements in detection platforms while acknowledging persistent challenges in real-world integration and standardization. We conclude that optimizing protein modification-based sensing strategies represents a crucial pathway for developing robust, clinically translatable diagnostic tools, and propose focused research directions to address existing technical barriers and accelerate practical implementation.

## 1. Introduction

Protein modifications, particularly post-translational modifications (PTMs), represent a fundamental biological phenomenon wherein proteins undergo covalent alterations after their synthesis. To clarify the scope of this review, while our primary focus is on classical enzymatic PTMs—such as phosphorylation, ubiquitination, glycosylation, methylation, SUMOylation, acetylation, crotonylation, and redox modifications—we also selectively include certain non-enzymatic covalent modifications (e.g., formaldehyde-derived adducts) and RNA modifications (e.g., m^6^A, m^5^C). This broader scope is justified by their growing relevance in regulatory biology and biosensing, their mechanistic parallels to PTMs, and their emerging roles as biomarkers detectable via similar sensing platforms. These modifications profoundly expand the functional repertoire and structural diversity of the proteome beyond the genetic code, enabling proteins to modulate a wide array of physiological processes. PTMs encompass over 650 distinct types, each contributing uniquely to protein function. By altering protein conformation, stability, localization, activity, and interactions, PTMs serve as critical regulators of cellular signaling, metabolism, gene expression, and immune responses [1,2]. The dynamic and reversible nature of many PTMs allows cells to rapidly adapt to environmental cues and maintain homeostasis. Conversely, dysregulation of PTMs is implicated in the pathogenesis of numerous diseases, including cancers, neurodegenerative disorders, metabolic syndromes, cardiovascular diseases, and infectious diseases [1,3,4].

The biological significance of PTMs extends to diverse molecular mechanisms. For example, phosphorylation modulates enzyme activity and signal transduction cascades; ubiquitination governs protein degradation and trafficking; glycosylation influences protein folding and cell–cell communication; and methylation impacts chromatin remodeling and gene transcription [1,5]. Emerging PTMs such as crotonylation, succinylation, and lactylation have been identified with distinct functional roles, highlighting the complexity of the PTM landscape [5,6]. Notably, PTMs can act in concert and engage in intricate crosstalk, where one modification influences the addition, removal, or functional outcome of another. This hierarchical and combinatorial regulation adds an additional layer of biological control and complexity [7]. Advances in proteomic technologies, including mass spectrometry-based approaches, have facilitated the high-throughput identification and quantification of PTMs, enabling comprehensive profiling of modification patterns across physiological and pathological states [8,9].

In the context of disease diagnosis, PTMs hold considerable promise as biomarkers and therapeutic targets, as their altered patterns faithfully reflect disease onset, progression, and therapeutic response. The diagnostic utility of specific PTMs is evident across a spectrum of diseases. In oncology, for instance, aberrant phosphorylation and acetylation of histone proteins are hallmarks of epigenetic dysregulation and cancer development [3,10], while disease-specific alterations in protein glycosylation on cell surfaces and secreted proteins modulate tumor–immune interactions and serve as established biomarkers for various cancers and inflammatory disorders [11]. Beyond cancer, PTMs are implicated in the pathogenesis of neurodegenerative diseases, where modifications such as S-nitrosylation and oxidative PTMs contribute to protein misfolding and neuronal damage [12,13]. Similarly, in metabolic diseases like diabetes and cardiovascular disorders, PTMs exert precise control over key metabolic enzymes and signaling pathways, directly influencing pathophysiology and presenting opportunities for intervention [4,14,15]. Furthermore, the relevance of PTMs extends to infectious diseases, where host- or pathogen-mediated modifications of viral proteins can critically modulate infection cycles and immune evasion strategies, underscoring their diagnostic value [16].

Biosensing technologies have evolved as powerful tools for detecting PTMs and their associated proteins, facilitating early diagnosis and monitoring of diseases. Biosensors integrate a biorecognition element with a transducer to convert a biological interaction into a measurable signal. Among various biosensing platforms, optical biosensors, including surface plasmon resonance (SPR), evanescent wave-based sensors, and surface-enhanced Raman scattering (SERS) sensors, have gained prominence due to their label-free, real-time, and highly sensitive detection capabilities [17,18,19]. SPR biosensors, for example, have been extensively applied in medical diagnostics to analyze biomolecular interactions and detect clinically relevant analytes such as proteins, nucleic acids, viruses, and exosomes [17,20]. The integration of nanomaterials, such as graphene, carbon dots, and silk-based matrices, has further enhanced biosensor performance by improving biocompatibility, sensitivity, and stability [21,22,23].

Recent advancements also include the development of wearable, flexible, and implantable biosensors that enable continuous monitoring of physiological parameters, facilitating personalized healthcare and early disease detection [20,24,25]. The convergence of biosensing with machine learning and artificial intelligence is poised to revolutionize data analysis and interpretation, enabling more precise and rapid diagnostics [18,26]. Despite these advances, challenges remain in biosensor miniaturization, stability, specificity in complex biological matrices, and regulatory compliance for clinical translation [27,28]. Addressing these challenges is critical to fully realizing the potential of biosensors in medical diagnostics.

In summary, protein modifications are central to biological regulation and disease pathogenesis, with significant implications for diagnostics. The synergy between advances in understanding PTMs and biosensor technologies offers unprecedented opportunities for sensitive, specific, and rapid detection of disease biomarkers. Continued interdisciplinary research integrating proteomics, chemistry, materials science, and bioengineering will drive the development of next-generation biosensors, overcoming existing challenges and enabling transformative applications in medical diagnostics and personalized medicine.

## 2. Main Body

### 2.1. Types and Functions of Protein Modifications

#### 2.1.1. Phosphorylation

**Figure 1:**
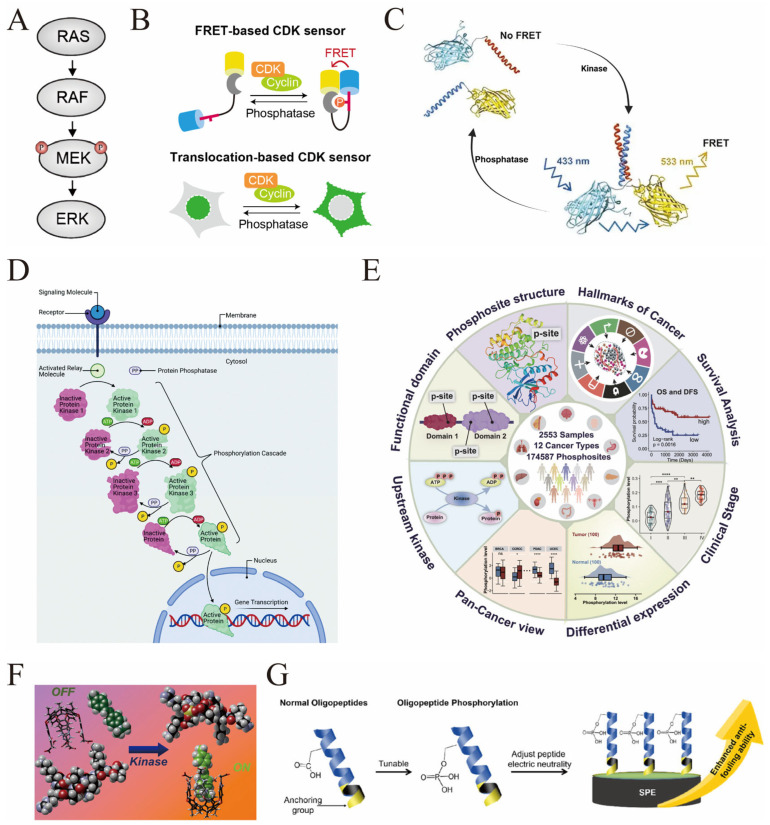
(**A**) The mitogen-activated protein kinase (MAPK) signaling pathway [reprinted with permission from ref. [29]. Copyright (2021) MDPI]. (**B**) This figure depicts two genetically encoded fluorescent biosensors (FRET-based and translocation-based) that enable real-time, single-cell monitoring of CDK activity in living cells by detecting phosphorylation-driven conformational changes or subcellular translocation, overcoming the limitations of traditional population-based assays and facilitating studies of spatiotemporal CDK regulation across biological systems, the above figure shows the fluorescent groups of the FRET (fluorescence resonance energy transfer) sensor (yellow and blue represent donor/receptor fluorescent molecules respectively); the red “P” indicates phosphorylation modification, reflecting the kinase activity of CDK. [reprinted with permission from ref. [31]. Copyright (2025) Japan Society for Cell Biology]. (**C**) This figure illustrates the mechanism of a phosphorylation-responsive FRET-based protein switch: in the “No FRET” state (left), the switch’s coiled-coil sensor domains (red/blue) and fluorescent actuator domains (cyan/yellow) are separated; kinase-mediated phosphorylation induces a conformational rearrangement (right), bringing the fluorescent domains into proximity to generate a FRET signal (excited at 433 nm, emitting at 533 nm), while phosphatase reverses this process—aligning with the study’s focus on PTM-driven protein switches that enable reversible, real-time sensing of kinase activity via dynamic FRET signal changes [reprinted with permission from ref. [32]. Copyright (2024) American Chemical Society]. (**D**) Illustration of a representative phosphorylation/dephosphorylation signal transduction pathway. Phosphorylation cascade transmission process of cellular signals: After extracellular signaling molecules bind to cell membrane receptors, the signals are transmitted into the cytoplasm via relay molecules. A cascade reaction is formed through the step-by-step phosphorylation of protein kinases (inactive pink kinases are modified into active green kinases). Meanwhile, protein phosphatases can reverse this process to regulate the signals. Finally, the activated proteins enter the nucleus to initiate gene transcription, completing the signal response from the extracellular environment to the nucleus, which reflects the dynamic regulatory role of phosphorylation in signal transmission [reprinted with permission from ref. [33]. Copyright (2021) The Royal Society of Chemistry]. (**E**) PhosCancer consolidates extensive phosphorylation site data (* Corresponding to *p* < 0.05, ** represents *p* value < 0.01, *** represents *p* value < 0.001, **** corresponding to *p* < 0.0001) [reprinted with permission from ref. [35]. Copyright (2024) Elsevier]. (**F**) “Switch” Sensing Process of the Host-Guest Molecular Pair: In the “OFF” state (left side), the host (black cavity structure) and the guest (green segment) remain in an unbound, signal-off state. After the intermediate kinase catalyzes the phosphorylation modification of the peptide, the “ON” state (right side) shows that the modified peptide forms a specific binding with the host, switching the sensing signal to the activated state. This clearly demonstrates the implementation of “OFF-ON” selective sensing for phosphorylated peptide modifications via the orthogonal recognition mechanism of the host–guest pair [reprinted with permission from ref. [37]. Copyright (2018) American Chemical Society]. (**G**) Inspired by the biomimetic process of protein phosphorylation, a peptide functionalized with a dihydrogen phosphate group (−PO_4_H_2_) was designed. Compared with natural and conventional peptides, this functionalized peptide exhibits significantly enhanced hydrophilicity and anti-fouling performance [reprinted with permission from ref. [39]. Copyright (2023) American Chemical Society].

Phosphorylation is a fundamental PTM involving the covalent attachment of a phosphate group, typically to serine, threonine, or tyrosine residues of proteins. This reversible modification, catalyzed by kinases and reversed by phosphatases, induces conformational changes that regulate protein activity, localization, stability, and interactions, thereby modulating diverse cellular processes. Mechanistically, phosphorylation alters the electrostatic properties and steric configuration of target proteins, enabling or disabling their interactions within signaling cascades. For instance, phosphorylation of kinase proteins triggers conformational rearrangements that activate or inhibit their catalytic functions, serving as molecular switches in signal transduction pathways. The mitogen-activated protein kinase (MAPK) pathway exemplifies this, where phosphorylation of MEK1 at specific residues (S218 and S222) by RAF kinases induces conformational changes essential for ATP binding and substrate phosphorylation, propagating oncogenic signals (Figure 1A) [29]. Advanced biosensor technologies, such as fluorescence resonance energy transfer (FRET)-based sensors, have been developed to monitor phosphorylation dynamics in living cells, providing spatiotemporal resolution of kinase activities and downstream signaling events (Figure 1B) [30,31]. Moreover, phosphorylation-driven protein switches have been engineered to respond rapidly and reversibly to kinase activity, facilitating real-time biosensing and synthetic biology applications (Figure 1C) [32]. These biosensors exploit the intrinsic conformational shifts induced by phosphorylation, underscoring the centrality of phosphorylation in cellular communication and its amenability to quantitative detection. The complexity of phosphorylation is further highlighted by proximal multi-site phosphorylation, where clusters of phosphosites cooperatively modulate protein conformation and function, influencing signal integration and gene expression (Figure 1D) [33]. Collectively, phosphorylation orchestrates signal transduction by modulating protein structure and interactions, and its dynamic regulation is critical for cellular homeostasis and response to environmental stimuli.

Aberrant phosphorylation patterns are hallmark features of numerous diseases, notably cancer, where dysregulated kinase activity drives oncogenic signaling, tumor progression, and metastasis. Phosphorylation serves as a sensitive biomarker reflecting the activation state of signaling pathways and the presence of pathological processes. For example, oncogenic mutations in kinases such as BRAF and MEK1 alter their phosphorylation states and conformations, which can be monitored using kinase conformation biosensors to evaluate drug efficacy and mutation-driven activity changes in live cells [29,34]. The development of phosphoproteomic databases like PhosCancer consolidates extensive phosphorylation site data across multiple cancer types, facilitating the identification of clinically actionable phosphosites and their associations with cancer hallmarks (Figure 1E) [35]. Biosensors leveraging phosphorylation-specific recognition elements enable sensitive detection of phosphorylated proteins, such as kinases and their substrates, in complex biological samples, offering rapid and minimally invasive diagnostic tools (Figure 1F) [36,37]. Nanostructured electrode platforms have been engineered to amplify electrochemical signals for detecting phosphorylated oncogenic proteins like BRAF with enhanced sensitivity, supporting their potential clinical application in cancer diagnostics [38]. Furthermore, phosphorylation of peptides and proteins can be exploited to design anti-fouling biosensor interfaces, improving detection accuracy in biological fluids, and molecular simulation (MS) illustrated that, compared with the -COOH and -NH_2_ groups, the -PO_4_H_2_ group formed the most numbers of hydrogen bonds and stronger hydrogen bonds with water molecules (Figure 1G) [39]. Following a similar logic, we hypothesize that some modifications that introduce strong polar and multi-hydrogen bond sites (such as glycosylation and hydroxylation) can also be used to enhance the hydrophilicity of natural peptides. In neurodegenerative diseases such as Alzheimer’s disease (AD), phosphorylated tau proteins (p-tau), particularly at specific residues like threonine 217 and 181, have emerged as robust biomarkers for early diagnosis and disease progression monitoring. Plasma and cerebrospinal fluid assays detecting p-tau isoforms demonstrate high specificity and sensitivity, outperforming traditional biomarkers and correlating with pathological hallmarks observed via PET imaging [40,41,42]. The pathological phosphorylation of tau also impairs its proteolytic degradation, contributing to biomarker accumulation and disease pathology [40]. Similarly, phosphorylated neurofilament heavy chains serve as biomarkers for neurodegeneration, reflecting axonal damage in diseases like amyotrophic lateral sclerosis [43]. In infectious diseases, phosphorylation patterns of viral proteins and host kinases reveal virus–host interactions and may guide antiviral strategies [44]. Collectively, phosphorylation-based biomarkers provide critical insights into disease mechanisms, enabling early detection, prognosis, and therapeutic monitoring across a spectrum of diseases, with biosensor technologies advancing their clinical translation.

#### 2.1.2. Acetylation

**Figure 2:**
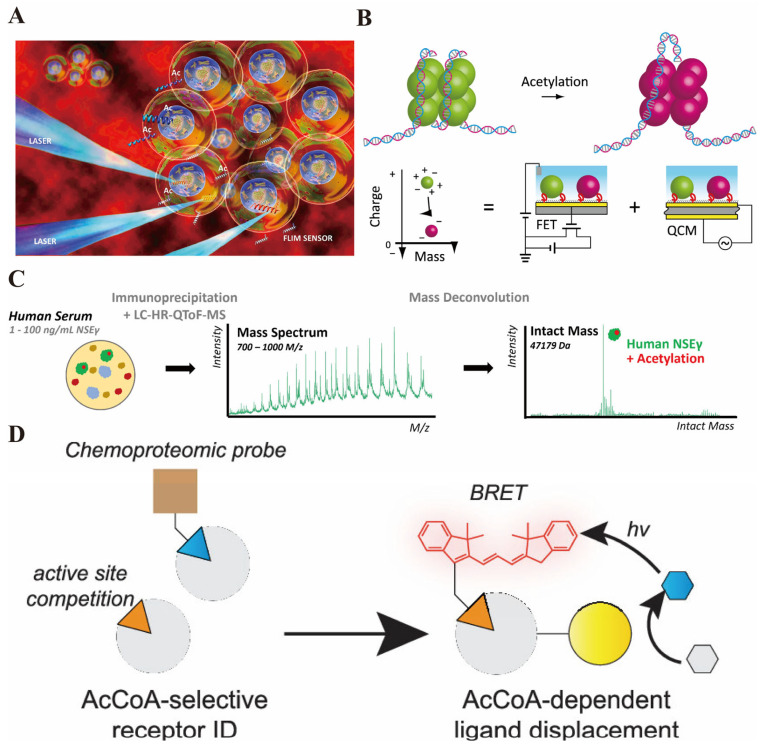
(**A**) The red background in the figure represents the cellular microenvironment. Multiple “microspheres” encapsulating internal structures simulate living cells. The peptide biosensor labeled as “Ac” is delivered into the cells. When stimulated by laser (LASER), the “FLIM SENSOR” combined with TCSPC-FLIM technology can detect the changes in the fluorescence lifetime of the sensor—this change corresponds to the histone acetylation process catalyzed by the acetyltransferase, thereby enabling real-time and high-spatial-resolution monitoring of the acetylation activity within living cells, which aligns with the core method described in the text: “Using peptide biosensors combined with FLIM technology to dynamically track the function of acetyltransferase” [reprinted with permission from ref. [46]. Copyright (2019) American Chemical Society]. (**B**) The schematic diagram shows the changes in histone acetylation—on the left, unacetylated histones (green) bind to DNA, and after acetylation, they transform into negatively charged histones on the right (pink), with both their charge and mass changing; below is the explanation of the detection logic: through the “charge–mass” coordinate axis, the acetylation leads to a decrease in the positive charge of histones and a change in mass, and this change can be detected separately by the integrated FET (field effect transistor) and QCM (quartz crystal microbalance) sensors—the FET captures the change in the charge of histones, and the QCM measures their adsorption mass, and finally, the “charge–mass ratio” is used to correlate the degree of histone acetylation [reprinted with permission from ref. [47]. Copyright (2020) MDPI]. (**C**) The analytical process for analyzing neuron-specific enolase γ (NSEγ) in human serum using immunoprecipitation combined with liquid chromatography–high-resolution quadrupole time-of-flight mass spectrometry (LC–HR-QToF-MS). First, the target protein was enriched from human serum containing 1–100 ng/mL neuron-specific enolase γ (NSEγ) through immunoprecipitation, and then analyzed by LC–HR-QToF-MS to obtain the mass spectrum (below); after quality deconvolution processing, the complete protein mass information of NSEγ was obtained (above), in which its acetylation modification form could be detected [reprinted with permission from ref. [49]. Copyright (2024) American Chemical Society]. (**D**) This image illustrates the Acetyl-CoA (AcCoA) sensing strategy developed based on chemical proteomics: On the left, the chemical proteomic probe competes with the active site to verify that NAA50 is an “AcCoA-selective receptor” capable of distinguishing AcCoA from CoA; On the right, after fusing NAA50 with Nanoluc fluorescent protein, when it binds to the fluorescent group (guest) connected to CoA, a BRET signal will be generated; And when AcCoA (the target metabolite) binds to NAA50 (the host), it will competitively replace the fluorescent group guest, achieving the detection of AcCoA through the change in BRET signal [reprinted with permission from ref. [51]. Copyright (2023) American Chemical Society].

Acetylation is a pivotal PTM involving the covalent attachment of an acetyl group, typically to lysine residues on histone and non-histone proteins. This modification profoundly influences protein function, stability, and interactions, thereby regulating diverse cellular processes. Central to gene expression regulation is histone acetylation, which neutralizes the positive charge on lysine residues within histone tails, diminishing histone–DNA interactions and resulting in a more relaxed chromatin structure. This chromatin decondensation facilitates the access of transcriptional machinery to DNA, promoting gene transcription. The dynamic balance between histone acetyltransferases (HATs) and histone deacetylases (HDACs) governs this process, enabling fine-tuned control of gene expression patterns essential for cellular differentiation, proliferation, and response to environmental cues. Beyond histones, acetylation modulates the activity and interactions of transcription factors, signaling molecules, and metabolic enzymes, thereby integrating epigenetic regulation with broader cellular metabolic states. For instance, acetyl-Coenzyme A (acetyl-CoA), a central metabolite, serves as the acetyl donor in acetylation reactions, linking cellular metabolism to epigenetic control. Recent advances have enabled the development of genetically encoded fluorescent biosensors capable of visualizing acetyl-CoA dynamics in live cells, revealing its compartmentalized metabolism and rapid fluctuations that may influence acetylation-dependent gene regulation (Figure 2A) [45,46]. Furthermore, acetylation plays critical roles in pathological contexts such as cancer and neurodegenerative diseases, where aberrant acetylation patterns disrupt normal gene expression and cellular homeostasis. The integration of acetylation with other PTMs, such as methylation and phosphorylation, contributes to the complexity of the epigenetic landscape. Thus, acetylation constitutes a versatile and dynamic regulatory mechanism that modulates chromatin architecture and gene expression, with profound implications for cellular function and disease.

The clinical utility of acetylation as a biomarker and diagnostic target has gained increasing attention due to its direct involvement in gene regulation and disease pathogenesis. Detection of acetylation modifications, particularly histone acetylation states, offers insights into cellular epigenetic status and disease progression at early stages. Innovative biosensing technologies have been developed to monitor acetylation with high sensitivity and specificity. For example, aptamer-functionalized field effect transistors (FETs) combined with quartz crystal microbalance (QCM) sensors enable label-free, real-time detection of histone acetylation by quantifying changes in charge and mass upon acetylation of histone subunits, achieving detection limits below 100 nM (Figure 2B) [47]. This approach facilitates early diagnosis by allowing precise measurement of PTM alterations implicated in diseases such as cancer. Additionally, CRISPR-Cas systems have been engineered as switchable biosensors responsive to acetylation states; a switchable Cas12a system has been employed to detect histone deacetylase (HDAC) activity with sub-nanomolar sensitivity, enabling direct biochemical assays for enzyme function relevant to epigenetic dysregulation [48]. Intact mass spectrometry combined with immunoaffinity purification has also been utilized to identify acetylated protein biomarkers, such as acetylated neuron-specific enolase, in human serum, highlighting its potential in tumor diagnosis (Figure 2C) [49]. Moreover, SPR biosensors have been applied to study acetylation-related enzyme–substrate interactions, providing label-free, real-time monitoring under near-physiological conditions, which is valuable for both diagnostics and drug development [50]. The modulation of protein–ligand interactions by acetylation has also been explored using advanced biosensor platforms. For instance, the Acetyl-CoA (AcCoA) sensing strategy developed based on chemical proteomics can utilize chemical proteomics to screen for receptors and construct a bioluminescence resonance energy transfer (BRET) sensing system, enabling the selective detection of AcCoA. (Figure 2D) [51]. Collectively, these biosensing modalities harness the biochemical specificity of acetylation modifications to enable early detection, monitoring, and therapeutic assessment in various diseases, underscoring acetylation’s critical role as a biomarker and diagnostic target.

#### 2.1.3. Glycosylation

**Figure 3:**
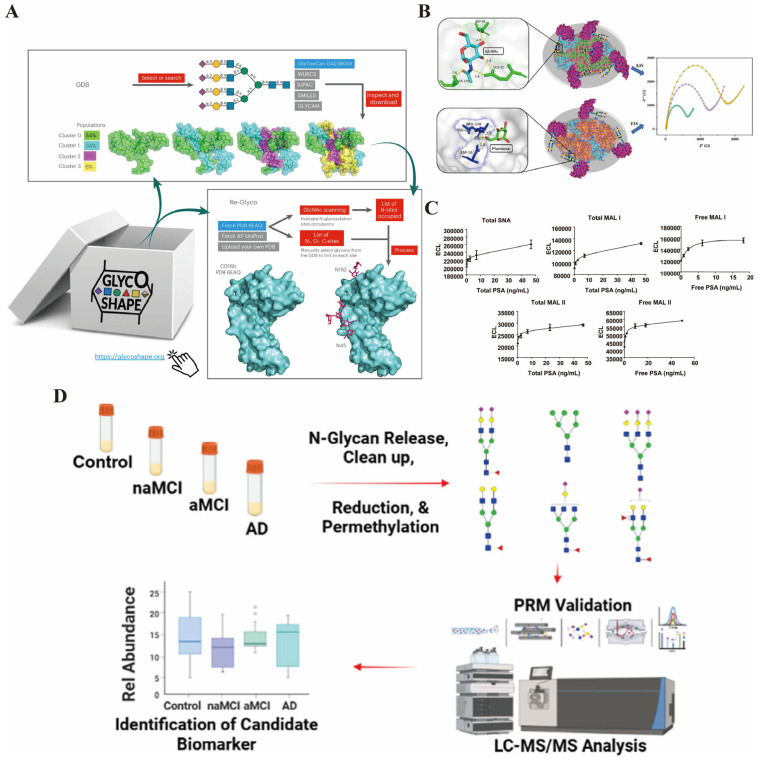
(**A**) the GlycoShape GDB is a repository of glycan 3D structures from 1 ms cumulative sampling through uncorrelated replicas of deterministic MD simulations [reprinted with permission from ref. [58]. Copyright (2024) Springer Nature]. (**B**) The WGA@MIL-101(Fe)-based biosensor demonstrates superior AFP sensing performance, attributed to the facile recognition of GlcNAc in the glycan chain. Concurrently, impedance data offers electrochemical evidence for the biantennary N-glycan structure of AFP [reprinted with permission from ref. [60]. Copyright (2025) American Chemical Society]. (**C**) The response characteristics of serum PSA glycosylation detection based on lectins: The five subgraphs in the figure correspond to five lectins (SNA, MAL I, MAL II) that form an immunoabsorption detection system. The content of different glycosylation forms of PSA is reflected by the electrochemiluminescence (ECL) signal [reprinted with permission from ref. [61]. Copyright (2009) American Chemical Society]. (**D**) The process for screening AD-related biomarkers based on N-glycan analysis: Firstly, the serum samples of normal cognition (Control), non-amnestic MCI (naMCI), amnestic MCI (aMCI), and AD subjects were subjected to N-glycan release, purification, reduction, and full methylation treatment to obtain different structures of N-glycans; then, through LC–MS/MS analysis combined with PRM verification, the differentially expressed N-glycans among the groups were identified; finally, the bar chart presented the relative abundance differences in candidate biomarkers in the four groups of samples [reprinted with permission from ref. [67]. Copyright (2025) American Chemical Society].

Glycosylation, a complex and highly diverse PTM, involves the covalent attachment of carbohydrate moieties (glycans) to proteins, resulting in glycoproteins that are critical for a wide array of biological functions. This diversity arises from variations in glycan composition, branching, linkage types, and site-specific occupancy, which collectively generate a heterogeneous glycan landscape on protein surfaces. For instance, N-linked glycosylation, where glycans attach to asparagine residues, can produce thousands of distinct glycoforms due to differences in monosaccharide composition, sequence, and three-dimensional conformation, contributing to both macro- and microheterogeneity at glycosylation sites [52]. Chao et al. designed a method that utilizes photo-degraded amino-modified graphene to covalently bind with the N-glycans in the serum glycoproteins of patients with liver cell carcinoma (HCC). This approach was used to capture the N-glycans. They found that there were significant differences in the N-glycan spectra between the serum samples of liver cancer patients and healthy individuals. These differences were manifested as an increase in the abundance and molecular weight of N-glycans. Through biomarker screening, an N-glycan with a *m*/*z* ratio of 1847.01 showed potential as a diagnostic biomarker for liver cancer [53]. The structural complexity of glycans enables them to mediate specific molecular recognition events essential for cell–cell communication, immune responses, and pathogen–host interactions. Glycans on cell surfaces act as recognition elements for lectins and other glycan-binding proteins, facilitating processes such as cell adhesion, signaling, and immune modulation [54]. Moreover, glycosylation patterns are tightly regulated and can change dynamically in response to physiological or pathological stimuli, influencing protein folding, stability, and trafficking [55]. The role of glycosylation in cell recognition is exemplified by its involvement in immune evasion by pathogens, where altered glycan structures mask antigenic epitopes or mimic host glycans to avoid detection [56]. Similarly, in cancer, aberrant glycosylation modifies cell surface properties, affecting metastasis and tumor–immune interactions [57]. Advances in analytical technologies, such as mass spectrometry and glycan databases like GlycoShape, have enhanced the ability to characterize glycan structures and their spatial orientation on proteins, providing insights into their functional roles in cellular recognition (Figure 3A) [58]. Additionally, engineered systems such as yeast surface display have been developed to study glycosylated proteins in a controlled manner, enabling exploration of glycan-mediated interactions [59]. Overall, the diversity of glycosylation underpins its central role in mediating specific and versatile cell recognition mechanisms critical for normal physiology and disease pathogenesis.

Glycosylation has emerged as a pivotal biomarker domain due to its sensitivity to pathological changes and its direct involvement in disease mechanisms. Aberrant glycosylation patterns on proteins have been extensively studied as diagnostic and prognostic indicators in various diseases, including cancer, autoimmune disorders, infectious diseases, and metabolic conditions. For example, altered glycosylation of alpha-fetoprotein (AFP) serves as a sensitive biomarker for hepatocellular carcinoma, where specific N-glycan structures such as terminal galactosylation and core fucosylation correlate with disease progression (Figure 3B) [60]. Similarly, prostate-specific antigen (PSA) glycosylation changes improve specificity in prostate cancer detection beyond total PSA levels, with biosensors capable of distinguishing glycoforms through lectin-based or aptamer-based impedimetric assays (Figure 3C) [61,62]. In neuroblastoma, serum protein N-glycosylation signatures, including decreased high-mannose and increased sialylated glycans, provide discriminatory power for early diagnosis [63]. The clinical utility of glycosylation biomarkers extends to autoimmune and inflammatory diseases, where genome-wide association studies have linked glycosyltransferase genes to disease susceptibility, highlighting glycosylation’s etiological relevance [64]. Technological innovations such as electrochemical biosensors leveraging lectin–glycan interactions enable rapid, sensitive, and cost-effective detection of glycoprotein biomarkers in complex biological samples, advancing point-of-care diagnostics [65,66]. Moreover, glycosylation-mediated biosensors have been developed for neurodegenerative diseases like Parkinson’s, detecting specific glycoproteins with high sensitivity using nanomaterial-based signal amplification (Figure 3D) [67]. The integration of glycosylation analysis with multi-omics approaches and advanced mass spectrometry techniques facilitates comprehensive profiling of glycan alterations, enabling structure-specific glycoproteomics that elucidate disease-associated glycan changes with high resolution [52,59]. Despite these advances, challenges remain in standardizing glycan biomarker assays and translating them into routine clinical practice. Nonetheless, the growing understanding of glycosylation’s role in disease and the development of sophisticated biosensing platforms underscore its promising potential as a rich source of biomarkers for early diagnosis, prognosis, and therapeutic monitoring across diverse medical fields.

#### 2.1.4. Emerging and Metabolism-Linked PTMs: Succinylation and Lactylation

Beyond the classical modifications, recent advances have uncovered a growing family of PTMs directly derived from cellular metabolites, such as succinylation and lactylation. Lysine succinylation, involving the transfer of a succinyl group from succinyl-CoA, introduces a significant negative charge and alters protein function in metabolism and stress response. Lysine lactylation, derived from lactate, has emerged as a novel histone mark linking cellular metabolism to gene regulation in immune function and tumor microenvironments [68]. Their detection, however, presents distinct challenges. While primarily detected by mass spectrometry or specific antibodies in research, their integration into biosensors is an emerging frontier. Pioneering strategies are exploring engineered reader domains, synthetic antibodies, or chemical probes specific for succinyl- or lactyl-lysine motifs. For instance, recent work on developing lactylation-specific chemical probes and affinity reagents for succinylation lays the groundwork for future electrochemical or optical biosensing platforms. The development of robust, selective recognition elements for these metabolism-linked PTMs represents a key opportunity for engineered protein tools, aiming to enable real-time monitoring of metabolic states and associated diseases through point-of-care diagnostics [69].

### 2.2. Advances in Biosensing Technology

#### 2.2.1. Electrochemical Sensors

Electrochemical sensing platforms offer powerful tools for detecting post-translational modifications (PTMs) due to their ability to transduce specific biochemical interactions into measurable electrical signals. The fundamental principle involves modifying electrode surfaces with recognition elements—such as antibodies, aptamers, or molecularly imprinted polymers (MIPs)—that are engineered to selectively bind PTM motifs (e.g., phosphate or glycan groups), thereby altering interfacial electrochemical properties. These changes are detected via techniques including cyclic voltammetry (CV), differential pulse voltammetry (DPV), electrochemical impedance spectroscopy (EIS), or amperometry. Key advantages for PTM detection include high sensitivity toward low-abundance modifications, rapid response, and compatibility with point-of-care formats. The incorporation of nanomaterials such as gold nanoparticles, graphene, carbon nanotubes, and MXenes further enhances conductivity and electrocatalytic activity, improving both specificity and detection limits for modified proteins [70,71,72].

However, PTM detection introduces unique challenges. Surface fouling from nonspecific adsorption remains a critical issue, particularly given the low stoichiometry of many PTMs in complex samples. Mitigation strategies include physical and chemical surface modifications using nanostructured coatings, antifouling polymers (e.g., PEG, zwitterionic polymers), and biomimetic membranes [73,74]. Reproducible functionalization of electrodes with PTM-specific receptors is also essential; pretreatment methods such as cyclic voltammetric sweeping and plasma treatments improve layer stability and uniformity [75]. The multi-step fabrication and modification processes required for PTM recognition can further hinder scalability and reproducibility. Advances in nanomaterial integration and surface chemistry are steadily addressing these issues, improving the viability of electrochemical sensors for PTM-based clinical diagnostics [76,77].

Several recent studies demonstrate the application of electrochemical sensors for PTM detection. MIP-based sensors have been designed with cavities complementary to PTM sites, enabling label-free and highly selective detection. For example, a sensor targeting phosphorylated milk amyloid A (MAA) incorporated a nanocomposite of reduced graphene oxide and gold nanoparticles, achieving a detection limit of 5 pg/mL through electropolymerization around the phosphoprotein template [78]. An enzymatic approach was also used to remove unmodified β-lactoglobulin from MIP sensors, enhancing selectivity toward glycosylated forms of the allergen in milk with a detection limit of 3.58 ng/mL [79].

**Figure 4:**
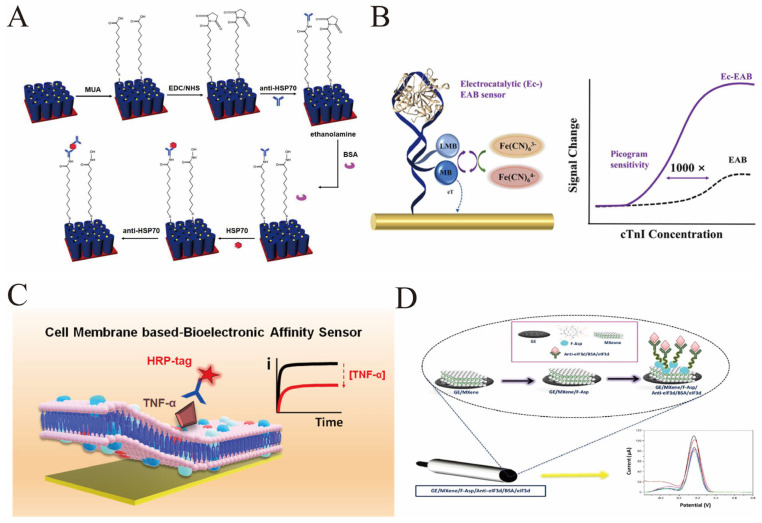
(**A**) Schematic procedure of covalent functionalization of the AgNPs/TNTs biosensor for the determination of HSP70. This figure illustrates the functionalization process of the AgNPs/TNTs biosensor for HSP70 detection: First, AgNPs/TNTs are incubated with MUA for modification, then activated with EDC/NHS to fix the anti-HSP70 antibody, and subsequently, the non-specific sites are blocked with ethanolamine and BSA; During the detection process, the HSP70 antigen is first bound, and then the anti-HSP70 antibody is bound again to form a sandwich complex. After each step, non-specific adsorption is washed away, and finally, the detection of different concentrations of HSP70 is achieved [reprinted with permission from ref. [80]. Copyright (2021) MDPI]. (**B**) On the left is the sensor structure: the aptamer probe is connected to methylene blue (MB) which is fixed on the electrode, and Fe(CN)_6_^3−^ in the solution acts as a catalytic redox reporter molecule; when cTnI binds to the aptamer, it will change the diffusion behavior of Fe(CN)_6_^3−^, thereby catalyzing the charge transfer process of MB and achieving signal amplification. The curve on the right compares the response of Ec-EAB and the traditional EAB sensor: the signal of Ec-EAB significantly increases with the increase in cTnI concentration, with a sensitivity reaching picogram level (limit of detection 10 pg/mL), which is 1000 times higher than that of the traditional EAB—this corresponds to the core design of “regulating the target-induced catalytic mechanism to achieve ultra-sensitive detection of cTnI while simplifying the preparation process”, meeting the requirements for rapid, reagent-free, and highly sensitive detection in the early diagnosis of acute myocardial infarction [reprinted with permission from ref. [82]. Copyright (2024) American Chemical Society]. (**C**) The sensor is modified with an electrochemical transducer using natural cell membranes (including macrophage membranes and red blood cell membranes) as the recognition layer. The natural receptors on the macrophage membranes can specifically capture the target antigen TNF-α, while the red blood cell membranes prevent non-specific adsorption. During the detection process, after binding to the HRP-labeled TNF-α antibody, quantitative detection is achieved through the change in the electrochemical signal (the curve on the right side of the figure) with the concentration of TNF-α. This design simplifies the modification steps of traditional immune sensors and achieves a low detection limit of 150 pM [reprinted with permission from ref. [74]. Copyright (2022) American Chemical Society]. (**D**) An electrochemical sensor based on MXene and aspartic acid-functionalized fullerene alcohol (F-Asp) for the selective and sensitive detection of the protein biomarker eIF3d. Firstly, MXene is modified on the surface of graphite electrode (GE), then fullerol functionalized with aspartic acid (F-Asp) is loaded. Subsequently, the anti-eIF3d antibody is fixed by the EDC/NHS chemical method (and is blocked with BSA), finally forming a sensing interface of GE/MXene/F-Asp/Anti-eIF3d/BSA. When eIF3d binds, quantitative detection is achieved through the change in electrochemical signals (current–voltage curve in the figure). The linear range of this sensor is 10–250 ng/mL, and the detection limit is as low as 0.14 ng/mL [reprinted with permission from ref. [85]. 2025; Copyright (2025) American Chemical Society].

Nanomaterial-enhanced electrodes have been particularly effective for PTM detection. Composites of gold nanoparticles and reduced graphene oxide improve electron transfer and provide ample surface area for immobilizing PTM-specific bioreceptors. A sensor for heat shock protein 70 (HSP70) modified with phospho-specific antibodies used titanium dioxide nanotubes and silver nanoparticles to achieve a linear detection range of 0.1–100 ng/mL (Figure 4A) [80]. A separate immunosensor for connective tissue growth factor (CTGF), built on nitrogen-doped graphene and targeting its glycosylated form, attained a detection limit of 0.0424 pg/mL and was validated in clinical serum samples [81].

Advanced catalytic mechanisms have also been integrated into electrochemical sensors to enhance sensitivity. For example, the Ec-EAB sensor alters the diffusion behavior of Fe(CN)_6_^3−^ and the mechanism of catalyzing the charge transfer of methylene blue through the binding of cTnI to the appropriate ligand. This enables highly sensitive detection of cTnI. The signal amplification effect increases the sensitivity by 1000 times compared to the traditional EAB (with a detection limit of 10 pg/mL) (Figure 4B) [82]. Quantum mechanical tunneling probes modified with redox cycling capabilities have pushed detection limits to the sub-picomolar range for viral proteins, showcasing the potential for ultra-sensitive diagnostics [83].

Aptamer-based electrochemical sensors have gained traction due to aptamers’ high specificity and stability. A truncated multi-thiol aptamer sensor for SARS-CoV-2 spike protein achieved an ultralow detection limit of 8.0 fg/mL and demonstrated variant selectivity, including enhanced sensitivity for the Omiron variant, enabling rapid and cost-effective point-of-care diagnostics [84]. Moreover, the use of natural cell membranes as recognition layers in bioelectronic sensors has been introduced to combine selective protein capture with antifouling properties, achieving detection limits as low as 150 pM for tumor necrosis factor alpha (TNF-α) (Figure 4C) [74].

Surface engineering of novel materials such as MXenes combined with functionalized fullerenols has also been reported for cancer biomarker detection, delivering detection limits as low as 0.14 ng/mL for eIF3d protein and demonstrating high selectivity in synthetic serum samples (Figure 4D) [85]. Additionally, electrochemical sensors based on nanostructured mesoporous gold electrodes have enabled the detection of protein phosphorylation, a critical post-translational modification in cancer, with enhanced signal amplification and sensitivity [38].

These examples collectively illustrate the diverse strategies employing protein modifications and nanomaterial enhancements in electrochemical sensors, addressing challenges of sensitivity, selectivity, and antifouling, and highlighting their growing utility in biomedical diagnostics and disease monitoring.

While these studies report impressively l ow detection limits, a critical consideration for their translational potential is the rigorous demonstration of reproducibility and robustness. The cited proof-of-concept studies typically establish performance under optimized laboratory conditions. For instance, the MAA sensor [86] demonstrated a low coefficient of variation (CV) for intra-assay measurements, and the β-lactoglobulin sensor [87] showed good recovery rates in spiked milk samples. However, comprehensive validation parameters such as inter-assay CV across different sensor batches, long-term stability, and performance in a large number of real clinical or field samples across multiple sites (multi-center studies) are essential future steps for these technologies. Such extensive validation is common in the later stages of biosensor development but is less frequently covered in initial feasibility studies. Therefore, while the reported LODs highlight the fundamental sensitivity achievable, their transition to reliable point-of-care diagnostics necessitates further work focused on standardization, reproducibility testing, and validation against gold-standard methods in diverse and complex sample matrices.

#### 2.2.2. Optical Sensors

**Figure 5:**
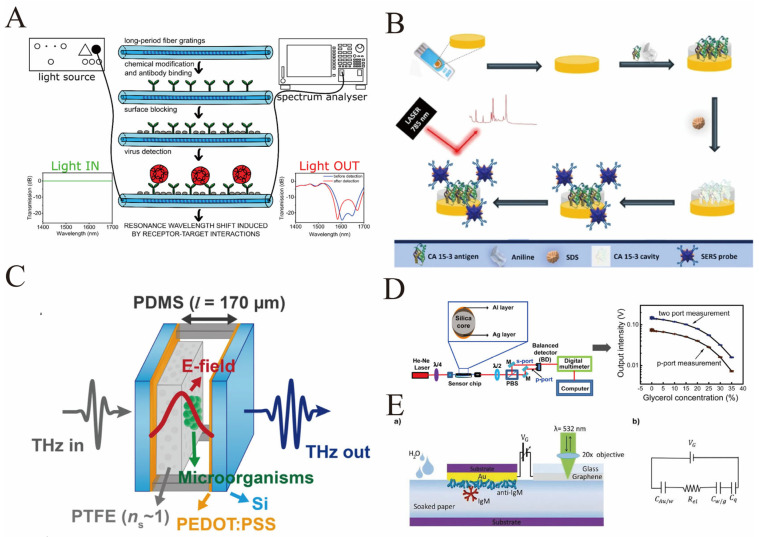
(**A**) The biosensor process for norovirus detection based on long-period fiber grating (LPFG): Firstly, the LPFG is chemically modified and the anti-VP1 antibody is fixed on it. After surface sealing, it is connected to the detection system of “light source–spectrometer”. When the norovirus VLPs bind to the antibody, it will induce a shift in the resonance wavelength. By comparing the light transmission spectra before and after the detection (blue and red curves), the binding event can be monitored in real time [reprinted with permission from ref. [88]. Copyright (2020) MDPI]. (**B**) Detection process of CA 15−3 (a biomarker for breast cancer) based on molecular imprinting polymers (MIPs) and SERS: Firstly, on a gold electrode, MIPs (MPan) are prepared by electro-polymerization using CA 15−3 as the template and aniline as the monomer. Then, the template is washed with SDS to obtain the CA 15−3 imprinted cavity. At the same time, gold nanostars (AuNSs) coupled with antibodies and Raman reporter molecules are prepared as SERS probes. During the detection process, after CA 15−3 binds to the imprinted cavity of MPan, the AuNSs probe is captured through the antibody–antigen interaction and generates enhanced Raman signals upon excitation by a 785 nm laser, thereby achieving highly sensitive detection of CA 15−3 in serum (linear range: 0.016–248.51 U mL^−1^) [reprinted with permission from ref. [92]. Copyright (2024) Springer Vienna] (**C**) Microbial detection sensor structure based on terahertz (THz) Fabry–Perot (FP) cavity: The sensor is supported by a porous PTFE film (with a dielectric constant close to 1), and an FP cavity is constructed by combining PDMS, Si, and PEDOT:PSS. At the center of the cavity, there is a strong terahertz electric field (E-field) due to the fundamental mode. When microorganisms enter the center of the cavity, they will change the transmission characteristics of the terahertz waves. The real-time detection of microorganisms is achieved by detecting the changes in the input/output terahertz signals—this design utilizes the strong electric field at the center of the cavity to enhance sensitivity [reprinted with permission from ref. [94]. Copyright (2021) MDPI]. (**D**) The surface plasmon resonance optical sensor system based on silver–aluminum bimetallic film optical fibers measures the difference in the intensity of p and s polarized light through the balanced zero-difference detection method, enabling high-resolution detection of the refractive index of glycerol solutions. It also has the advantages of miniaturization and high stability, and can be extended to biomedical detection [reprinted with permission from ref. [97]. Copyright (2020) American Chemical Society]. (**E**) (**a**) Schematic cross-sectional view of the sensing device, which includes an Au electrode biofunctionalized with an anti-IgM layer comprising 1012 molecules cm^−2^, and a graphene electrode capacitively coupled via a water-soaked paper strip. An external DC voltage (VG) is applied between the Au and the graphene electrodes by a voltage generator. (**b**) Equivalent circuit of the device, including the capacitances of the EDLs at the Au/water (CAu/w) and water/graphene (Cw/g) interfaces, the quantum capacitance of graphene (Cq) and the resistance of the water strip (Rel). The Raman spectrum of graphene is recorded by a micro-Raman spectrometer in a backscattering configuration, using a 532 nm laser [reprinted with permission from ref. [98]. Copyright (2025) Wiley-Blackwell].

Optical sensors have become a cornerstone in the detection of protein modifications due to their high sensitivity, real-time monitoring capabilities, and potential for label-free analysis. These sensors exploit various optical phenomena such as fluorescence, SPR, evanescent wave interactions, and refractive index changes to transduce biochemical interactions into measurable signals. The diverse types of optical sensors include fiber-optic biosensors, fluorescence-based probes, SERS sensors, and microstructured optical fiber (MOF) sensors, each with unique advantages tailored to specific applications. Fiber-optic sensors, particularly those based on long-period fiber gratings (LPFGs) or tilted fiber Bragg gratings (TFBGs), have demonstrated exceptional sensitivity and specificity in detecting protein modifications by monitoring changes in refractive index or binding-induced spectral shifts. For instance, LPFGs functionalized with antibodies have enabled label-free detection of viral proteins such as norovirus and SARS-CoV-2 spike protein, showcasing their utility in rapid diagnostics with minimal sample preparation (Figure 5A) [88,89]. The integration of functional nucleic acids like aptamers into fiber-optic evanescent wave (FOEW) sensors has further expanded the detection repertoire to include a broad range of biomolecules, enhancing specificity and enabling continuous monitoring [90]. Fluorescent probes, including boron dipyrromethene (BODIPY)-based fluorophores, have been effectively employed to detect pathological protein aggregates in neurodegenerative diseases such as Alzheimer’s, offering advantages in photostability and cell permeability for in vivo imaging [91]. SERS-based sensors leverage localized surface plasmon resonance in metallic nanostructures to achieve extraordinary signal enhancement, enabling ultrasensitive detection of cancer biomarkers like CA 15-3 with the aid of molecularly imprinted polymers for selective recognition (Figure 5B) [92]. Additionally, advances in microstructured optical fibers have facilitated enhanced refractive index sensitivity through tailored fiber modifications and surface functionalization, making them ideal for biochemical analyses requiring small sample volumes and low analyte concentrations [93]. Recent developments also include innovative sensor designs such as microbubble optofluidic channels integrated within Fabry-Pérot cavities, achieving ultrahigh refractive index sensitivity without the need for probe modification, thus enabling real-time and multiplexed biomolecular detection (Figure 5C) [94]. Collectively, these optical sensor platforms demonstrate remarkable versatility and sensitivity in detecting protein modifications, with ongoing research focused on improving stability, specificity, miniaturization, and integration for point-of-care and in vivo applications.

The practical application of optical sensors for detecting protein modifications has seen significant progress, particularly in the context of disease diagnostics and biomarker monitoring. One notable example involves the use of LPFG-based immunosensors for the detection of viral proteins such as norovirus and SARS-CoV-2 spike protein. These sensors utilize antibody immobilization on fiber surfaces to capture target proteins, inducing refractive index changes that shift the resonance wavelengths, enabling label-free, rapid, and sensitive detection with limits reaching nanogram per milliliter levels [88,89]. In neurodegenerative disease diagnostics, fluorescent aptasensors have been developed for tau protein detection, employing aptamer probes combined with biolayer interferometry to achieve selective and real-time monitoring in complex biological fluids, highlighting the potential for early-stage disease diagnosis [95]. Similarly, BODIPY-based fluorescent probes have been tailored to detect amyloid-beta plaques and neurofibrillary tangles in Alzheimer’s disease, capitalizing on their photostability and modifiable optical properties for in vitro and in vivo imaging [91]. Fiber loop ringdown spectroscopy (FLRDS) combined with fiber optic sensors has been innovatively applied to monitor beta-amyloid protein fragments, demonstrating ultra-sensitive detection down to parts per million concentrations in artificial cerebrospinal fluid, which is promising for early Alzheimer’s disease detection [96]. SPR sensors with bimetallic thin films and homodyne balanced detection methods have enhanced sensitivity for detecting protein interactions such as C-reactive protein antibody conjugates, offering small-sized, stable, and cost-effective platforms suitable for biomedical assays (Figure 5D) [97]. Furthermore, SERS sensors combined with molecularly imprinted polymers have enabled selective detection of cancer biomarkers like CA 15-3 at ultralow concentrations, demonstrating the capability of optical methods to achieve high specificity and sensitivity in complex biological matrices [92]. The development of modification-free optical immunosensors utilizing microbubble optofluidic cavities has allowed real-time, dynamic monitoring of protein binding events without the need for chemical labeling, significantly simplifying assay protocols and enhancing sensor reusability [94]. Additionally, graphene-based opto-electronic platforms have pushed detection limits to zeptomolar concentrations for immunoglobulin M, integrating Raman spectroscopy with biofunctionalized surfaces to achieve unprecedented sensitivity for biomarker detection (Figure 5E) [98]. These examples collectively illustrate the broad applicability and continuous innovation in optical sensor technologies for detecting protein modifications, addressing challenges such as sensitivity, specificity, assay speed, and integration into portable diagnostic devices.

### 2.3. Application of Protein Modifications in Disease Diagnosis

#### 2.3.1. Early Diagnosis of Cancer

Protein PTMs have emerged as critical molecular events that reflect the dynamic state of cellular processes and are profoundly implicated in cancer pathogenesis. These modifications, including phosphorylation, methylation, acetylation, ubiquitination, glycosylation, and RNA modifications such as m6A and m5C methylation, alter protein function, stability, localization, and interactions, thereby influencing tumor initiation and progression. The aberrant patterns of these modifications in cancer cells provide a rich source of potential biomarkers for early cancer diagnosis. For instance, dysregulated phosphorylation of oncogenic kinases like Aurora A (AURKA) and BRAF has been exploited to develop biosensors capable of detecting their activated forms with high sensitivity, enabling early identification of malignant transformation [38,99]. Similarly, RNA modifications such as m6A and m5C have been increasingly recognized for their roles in tumorigenesis, with altered expression of m6A regulatory proteins (writers, erasers, readers) correlating with cancer progression in hepatocellular carcinoma, colorectal cancer, and gynecological malignancies [100,101,102]. These RNA modifications influence RNA stability, translation, and immune evasion, making them promising biomarkers and therapeutic targets.

**Figure 6:**
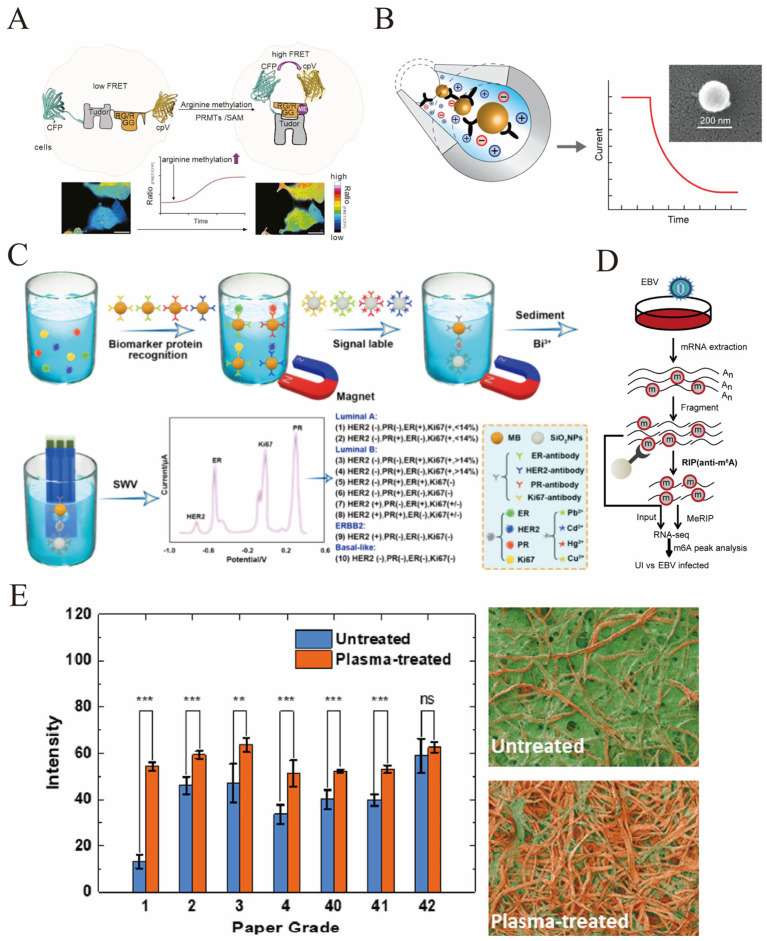
(**A**) The gene-encoded biosensor GEMS based on Förster resonance energy transfer (FRET) is used to quantitatively monitor the dynamic changes in arginine methylation (ArgMet) in live cells in real time [reprinted with permission from ref. [107]. Copyright (2024) Elsevier]. (**B**) The principle of unlabeled detection of sEV (exosomes) based on electrodynamics: The inner surface of the silicon microcapillary is fixed with affinity reagents targeting the surface markers of sEV. When sEV enters the capillary, its surface markers bind to the affinity reagents, altering the charge distribution within the capillary; by monitoring the decline in the flow current over time (the right curve), the detection of sEV can be achieved (the electron microscope image on the upper right of the figure shows the morphology of sEV) [reprinted with permission from ref. [109]. Copyright (2019) American Chemical Society]. (**C**) An electrochemical homogeneous biological platform based on metal ions/SiO_2_ nanoparticles/magnetic beads is used for simultaneous detection of four biomarkers of breast cancer (ER, PR, HER2, Ki67) and for achieving molecular classification of breast cancer [reprinted with permission from ref. [115]. Copyright (2024) American Chemical Society]. (**D**) Schematic representation of the MeRIP-seq protocol used to identify differential m6A modification following infection of HK1 and NP460 cells with EBV-GFP [reprinted with permission from ref. [127]. Copyright (2021) Elsevier]. (**E**) The optimization effect of plasma treatment on the protein adsorption performance of different grades of cellulose paper: The left column chart shows that for each grade of cellulose paper treated by plasma (orange columns), the protein adsorption signal intensity (Intensity) is significantly higher than that of the untreated group (blue columns, most of the differences are at the extremely significant level); The right microscopic structure image directly demonstrates the changes in the fibers before and after treatment—the surface of untreated paper fibers has an inert film, while after plasma treatment, the film is removed, exposing a fiber structure that is more conducive to protein binding (** represents *p* < 0.01, *** represents *p* < 0.001) [reprinted with permission from ref. [133]. Copyright (2023) American Chemical Society].

Protein glycosylation changes are also notable in ovarian and other cancers, where altered glycan structures on serum proteins can serve as early diagnostic indicators [103]. Ubiquitination and deubiquitination enzymes (DUBs) regulate protein turnover and have been linked to cancer cell proliferation and metastasis; their substrates and regulators are being explored as biomarkers in ovarian and breast cancers [104,105]. Moreover, novel PTMs such as N4-acetylcytidine (ac4C) in RNA and arginine methylation in proteins have been implicated in tumor progression and immune modulation, with emerging biosensors developed to detect these modifications in clinical samples (Figure 6A) [106,107].

The detection of autoantibodies against PTMs further enriches the biomarker landscape, as these humoral responses can precede clinical symptoms and provide early diagnostic clues [108]. Additionally, exosome-carried proteins bearing specific PTMs are being harnessed as non-invasive biomarkers for early cancer detection, with electrochemical biosensors developed to sensitively analyze multiple exosomal surface proteins such as EGFR, CEA, and EpCAM (Figure 6B) [109]. Keshavarz et al. proposed a non-chemically balanced Q-structured TiO_x_ template. Through a synergistic strategy of quantum-scale regulation and oxygen vacancy introduction, the SERS enhancement factor (EF) was increased to 3.4 × 10^7^, and the detection limit was reduced to 1 nM, achieving a sensitivity comparable to that of precious metals. This template can efficiently enhance the Raman signals of breast cancer-related biomarkers such as EGFR, and its clinical practicability has been verified in cancer cell lines [110]. The integration of PTM analysis with advanced biosensing technologies, including electrochemical, fluorescence, and nanomaterial-based sensors, has enhanced sensitivity and specificity, facilitating the detection of low-abundance cancer biomarkers in bodily fluids [111,112].

In summary, the study of protein and RNA modifications offers a multifaceted approach to cancer biomarker discovery. These modifications reflect the molecular heterogeneity and dynamic changes in tumor biology, providing valuable targets for early diagnosis. Continued advances in understanding the mechanisms and developing sensitive detection platforms are essential to translate these biomarkers into clinical practice, improving early cancer detection and patient outcomes.

The complexity and heterogeneity of cancer necessitate multiplexed detection platforms capable of simultaneously analyzing multiple biomarkers to improve diagnostic accuracy and enable personalized medicine. Recent advances in biosensor technology have facilitated the development of multiplex platforms that integrate detection of various protein modifications, nucleic acids, and extracellular vesicle components.

Electrochemical biosensors have gained prominence due to their high sensitivity, rapid response, and adaptability for multiplex analysis. For example, biosensors employing nanomaterials such as MXenes combined with gold nanoparticles have been engineered to detect multiple exosome surface proteins concurrently, enabling differentiation of tumor-derived exosomes from diverse cancer types (Figure 6B) [109]. Similarly, screen-printed electrodes (SPEs) functionalized with nanomaterials and biomolecules have been developed for low-cost, multiplex detection of breast cancer biomarkers, including proteins, genetic markers, and metabolites, offering potential for point-of-care applications [113].

Organic field-effect transistors (OFETs) functionalized with carbon dots have demonstrated remarkable sensitivity and selectivity for low-abundance proteins like carcinoembryonic antigen (CEA), a key tumor marker, highlighting the utility of nanomaterial-based platforms in multiplex biomarker detection [114]. Furthermore, homogeneous electrochemical biosensing strategies have been designed to simultaneously quantify multiple breast cancer markers such as ER, PR, HER2, and Ki67 in serum samples with high sensitivity and reduced assay time, facilitating rapid molecular typing and personalized treatment decisions (Figure 6C) [115].

Innovative biosensor designs also leverage protein or peptide engineering to create bifunctional complexes that enable simultaneous target recognition and signal reporting, enhancing multiplex detection capabilities [116]. Additionally, aptamer-based sensors targeting oncogenic proteins like PTK7 have been developed for multiplexed cancer cell recognition and early diagnosis [117].

The integration of these multiplex biosensing platforms with advanced signal amplification techniques, such as CRISPR/Cas systems, rolling circle amplification, and strand displacement amplification, further improves detection limits and assay robustness [118,119]. Moreover, the use of nanostructured electrodes, such as mesoporous gold and graphene-based materials, enhances electrochemical performance and enables the sensitive detection of multiple protein phosphorylation events and other PTMs relevant to cancer [38,71].

Overall, the development of multiplex biomarker detection platforms represents a significant advancement in cancer diagnostics. These platforms address tumor heterogeneity by providing comprehensive molecular profiles through simultaneous analysis of diverse biomarkers (Table 1). Continued innovation in biosensor design, nanomaterial integration, and signal amplification will be critical to achieving clinically viable multiplex diagnostic tools that enable early cancer detection, prognosis, and personalized therapeutic monitoring.

#### 2.3.2. Monitoring of Infectious Diseases

Protein PTMs, including glycosylation, phosphorylation, ubiquitination, and lipidation, play pivotal roles in viral infections by modulating both viral and host protein functions, thereby influencing viral entry, replication, immune evasion, and pathogenesis. Glycosylation, the enzymatic addition of glycans to proteins and lipids, is one of the most critical PTMs affecting viral infectivity and host immune recognition. For enveloped viruses, such as coronaviruses and herpesviruses, glycosylation of viral envelope proteins facilitates proper protein folding, stability, and mediates interactions with host cell receptors, enabling viral attachment and entry. For instance, the spike (S) protein of SARS-CoV-2 undergoes extensive N- and O-glycosylation, which not only assists in receptor binding but also shields immunogenic epitopes from neutralizing antibodies, contributing to immune evasion [120,121]. Similarly, avian infectious bronchitis virus (IBV), a coronavirus affecting poultry, exhibits conserved N-glycosylation and palmitoylation sites on its S1 protein, which are essential for viral infectivity and immune recognition; mutations in phosphorylation sites of this protein further modulate viral virulence and vaccine escape potential [122].

Phosphorylation also critically regulates host antiviral responses. The RNA sensor RIG-I, a key cytosolic pathogen recognition receptor, is modulated by phosphorylation and ubiquitination. Herpes simplex virus 1 (HSV-1) encodes the US3 kinase, which phosphorylates RIG-I at specific serine residues, suppressing its activation and downstream type I interferon responses, thus facilitating viral immune evasion [123]. This exemplifies how viruses exploit PTMs to subvert host immunity. Additionally, ubiquitination regulates immune signaling pathways and protein degradation during infection, influencing the balance between viral clearance and persistence [124]. Protein lipidation, such as palmitoylation and myristoylation, affects viral protein localization and function, impacting viral assembly and release [125,126]. Furthermore, emerging evidence highlights novel RNA modifications like N6-methyladenosine (m6A) that modulate viral and host transcript stability and translation, influencing viral replication and host responses, as seen in Epstein–Barr virus infection (Figure 6D) [127,128].

The dynamic interplay of these PTMs on viral and host proteins underscores their central role in the viral life cycle and pathogenesis. Advances in glycomics, proteomics, and epigenetic profiling technologies have enabled detailed characterization of these modifications, revealing their potential as biomarkers and therapeutic targets. For example, aberrant glycosylation patterns serve as diagnostic markers and vaccine targets, while modulation of phosphorylation or ubiquitination pathways offers avenues for antiviral drug development [129,130]. Understanding the mechanisms by which protein modifications regulate viral infection and host immunity is essential for developing innovative strategies to combat infectious diseases effectively.

Rapid, sensitive, and specific diagnostic tools are critical for effective infectious disease surveillance and control. Protein modifications have been harnessed to develop advanced biosensors and diagnostic platforms that enable early detection of viral pathogens and monitoring of disease progression. Glycosylation patterns on viral proteins and host receptors provide unique molecular signatures that can be exploited for biomarker discovery and biosensor design. For instance, aptamer-based biosensors targeting viral glycoproteins, such as the SARS-CoV-2 spike protein, have demonstrated remarkable sensitivity and variant specificity. A refined multi-thiol aptamer electrochemical biosensor exhibited ultralow detection limits (8.0 fg/mL) and enhanced selectivity for SARS-CoV-2 variants, including Omicron, outperforming conventional rapid tests [84]. Similarly, DNA aptamers against viral structural proteins like Enterovirus 71 VP1 have been developed for rapid chemiluminescence-based detection, combining high specificity with antiviral therapeutic potential [131].

Electrochemical label-free biosensors leveraging protein modifications offer ultrasensitive multiplex detection capabilities for infectious disease biomarkers, facilitating point-of-care diagnostics without complex sample preparation [132]. Nanomaterial-enhanced biosensors utilize the unique electrical and optical properties of nanostructures to improve protein adsorption and signal amplification, enabling rapid detection of pathogens in clinical and environmental samples (Figure 6E) [133,134]. Paper-based biosensors modified to optimize protein binding have been developed as cost-effective, portable platforms suitable for low-resource settings, addressing the global need for accessible diagnostics [133].

Emerging technologies such as CRISPR-based biosensors integrate nucleic acid recognition with protein-based signal transduction, offering high specificity and sensitivity for detecting viral RNA without amplification, thus simplifying workflows and reducing time-to-result [135]. Additionally, mRNA-LNP immunization combined with hybridoma technology accelerates monoclonal antibody production against viral proteins, enhancing the development of diagnostic reagents and therapeutic antibodies [136].

Wearable and implantable biosensors incorporating protein modification recognition elements enable continuous monitoring of infectious disease biomarkers and host immune responses, facilitating real-time health management and early outbreak detection [137,138]. Nanoplasmonic biosensors further expand diagnostic capabilities by detecting a wide range of biomolecules, including glycosylated proteins, with high sensitivity and environmental sustainability [139].

Overall, the integration of protein modification knowledge into biosensor design has revolutionized infectious disease diagnostics, offering rapid, accurate, and affordable tools essential for timely intervention and public health surveillance. Continued advancements in molecular engineering, nanotechnology, and bioinformatics are expected to further enhance the performance and accessibility of these diagnostic platforms, addressing current challenges such as variant detection, multiplexing, and field deployment [140].

#### 2.3.3. Single-Cell and Spatial Proteomic Technologies for Protein Modification Detection

Single-cell proteomics enables high-resolution analysis of protein expression and PTMs. Mass spectrometry (MS) directly quantifies thousands of proteins from individual cells without amplification [141]. Advanced MS instrumentation improves sensitivity and throughput for detecting low-abundance PTMs like phosphorylation [142]. Miniaturized automated preparation minimizes sample loss [143], while native MS expands characterization [144]. Microfluidics enables precise single-cell manipulation in integrated platforms, allowing high-throughput analysis of secreted proteins [145]. Droplet microfluidics supports single-cell encapsulation screening [146,147]. Integration with MS creates comprehensive workflows. Nanopore sensing offers label-free, real-time protein and PTM detection [148]. AI-assisted signal recognition enhances resolution [149]. Key challenges include limited sample amounts and inability to amplify proteins [150]. Reproducible detection of low-abundance PTMs remains difficult. Multiplexed PTM profiling requires specific enrichment, increasing sample consumption. Nanopore detection shows promise but needs throughput improvement [148,149]. Data analysis is complex due to sparse datasets. Machine learning methods are essential [138]. Multi-omics integration complicates interpretation but is promising [141]. Sample handling challenges affect sensitivity, requiring efficient isolation methods [145,150,151]. Applications reveal cancer heterogeneity through histone PTM profiling and elucidate immune cell states via phosphorylation/acetylation analysis [152]. AI integration enhances data interpretation [153]. Future developments include microfluidics–MS–nanopore integration for high-throughput proteomics [141] and spatial PTM mapping.

Spatial proteomics integrates transcriptomic and proteomic data to map tissue biomolecules. MS imaging (MALDI, DESI, SIMS) enables label-free PTM profiling, while multiplexed immunofluorescence (mIF) provides targeted detection. Their integration combines broad coverage with specificity [154]. Spatial PTM detection faces dynamicity and abundance challenges. MALDI-MSI enables untargeted localization, with expansion techniques improving resolution. mIF allows specific detection but is antibody-limited. Emerging labeling methods enhance sensitivity [155,156,157]. Integration with computational workflows improves analysis. Spatial PTM profiling reveals disease mechanisms and biomarkers, showing cellular heterogeneity in cancer and other diseases [158,159,160]. Multi-omics integration enables systems-level understanding. Clinical translation requires standardized protocols and cost-effective platforms. Future development needs single-cell resolution, enhanced multiplexing, and AI integration.

Protein modification detection has evolved to enable multiplexed in vivo monitoring. Single-cell techniques reveal cellular heterogeneity, while spatial proteomics enables tissue context mapping. These advances support precision medicine through improved disease understanding and biomarker discovery.

### 2.4. Food Safety and Environmental Monitoring

#### 2.4.1. Detection of Protein Modifications in Food

**Figure 7:**
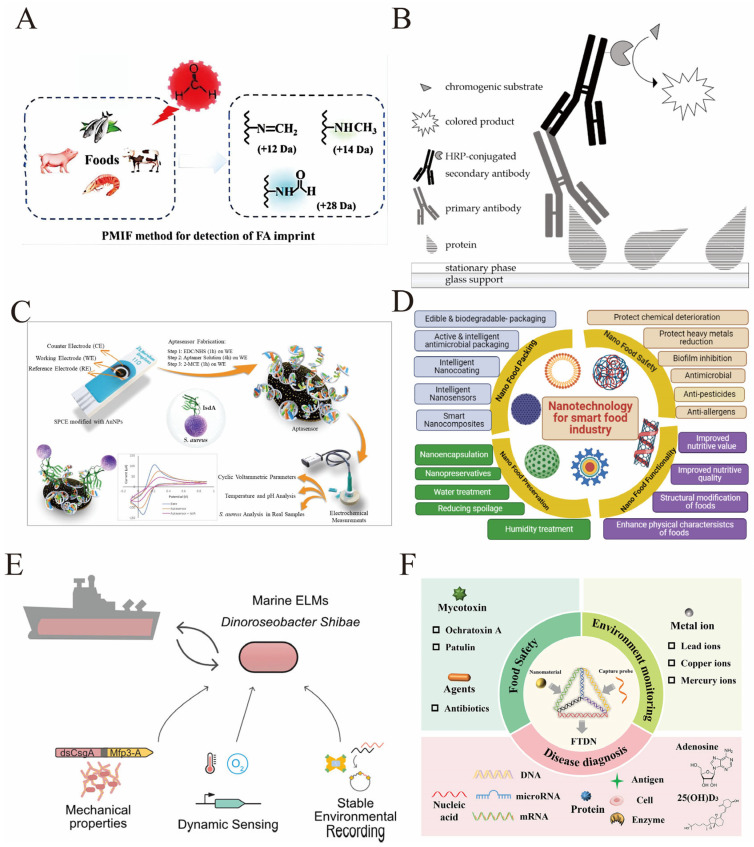
(**A**) The method based on protein formaldehyde modification (PMIF) is used to detect the residual traces of formaldehyde (FA) in food [reprinted with permission from ref. [161]. Copyright (2022) American Chemical Society]. (**B**) Schematic representation of the principle of an indirect antibody-based detection of proteins after chromatographic separation (HPTLC-IS). The primary antibody having affinity for the target protein is associated with the HRP-conjugated secondary antibody specific for the host of the primary antibody. Visualization of the ligated antibodies is achieved by the formation of a colored precipitate derived from a chromogenic substrate [reprinted with permission from ref. [162]. Copyright (2021) MDPI]. (**C**) The construction and characterization process of an electrochemical aptamer sensor for detecting *Staphylococcus aureus* (*S. aureus*) [reprinted with permission from ref. [171]. Copyright (2021) MDPI]. (**D**) The application of nanotechnology in the intelligent food industry [reprinted with permission from ref. [174]. Copyright (2023) MDPI]. (**E**) Carry out engineering modifications on the marine bacterium Dinoroseobacter shibae to transform it into an engineered living material suitable for marine applications [reprinted with permission from ref. [178]. Copyright (2025) American Chemical Society]. (**F**) The application of functionalized tetrahedral DNA nanostructures (FTDN) in the field of biosensing [reprinted with permission from ref. [183]. Copyright (2025) ELSEVIER].

The detection of protein modifications in food matrices plays a crucial role in ensuring food safety, authenticity, and allergen management. Protein modifications can arise naturally during processing or be induced by illicit additives, and their identification provides a molecular fingerprint for monitoring food quality and safety. For instance, formaldehyde (FA) is illegally added as a preservative in some food products, but its direct detection is challenging once the food is rinsed. A novel strategy based on permanent protein modifications induced by formaldehyde (PMIF) has been developed to detect FA addition in foods. By combining mass spectrometry with unrestrictive identification of protein modifications, four characteristic PMIFs were identified as markers for FA contamination, enabling detection even after thorough washing of food products (Figure 7A) [161]. This approach exemplifies how protein modifications serve as persistent indicators of chemical adulteration. Another application is in the detection of allergenic proteins in processed foods. Food processing can induce structural changes and covalent modifications in allergenic proteins, affecting their immunogenicity and detectability. For example, the conjugation of bioactive plant metabolites such as benzyl isothiocyanate (BITC) to whey protein α-lactalbumin alters its allergenic properties. BITC modification changes the protein’s secondary structure, surface hydrophobicity, and proteolytic stability, which can increase allergenicity due to exposure or formation of new epitopes (Figure 7B) [162,163]. Detecting such modifications is essential for protecting sensitized individuals and improving allergen labeling accuracy. Mass spectrometry techniques, particularly MALDI-TOF/TOF, have been extensively employed for proteomic analysis of food allergens, providing detailed characterization of protein modifications induced by processing. These methods facilitate the identification of allergenic proteins in complex food matrices such as milk, egg, hazelnut, and lupin seeds, supporting allergen detection and risk assessment [164,165]. Additionally, targeted mass spectrometry has been used to quantify processed soy protein residues in food products, overcoming limitations of antibody-based assays that are affected by protein modifications during processing [166]. Furthermore, protein modifications are exploited to detect food adulteration and contamination. For example, molecularly imprinted polymer nanogel-based fluorescence sensors have been developed for rapid detection of porcine serum albumin to monitor pork contamination in halal meat products, offering high sensitivity and selectivity [167]. Similarly, electrochemical aptasensors utilizing aptamer-protein interactions have been designed for detecting food allergens such as peanut proteins and gluten components, enabling rapid, sensitive, and portable food safety testing [168,169]. In summary, the application of detecting protein modifications in food safety encompasses identifying illicit chemical additives, characterizing allergenic proteins and their processing-induced modifications, and monitoring food adulteration. Advances in mass spectrometry, aptamer-based biosensors, and molecular imprinting technologies provide powerful tools for sensitive and specific detection, thereby enhancing food safety surveillance and consumer protection.

Emerging detection technologies based on protein modifications have significantly advanced food safety by enabling rapid, sensitive, and specific analysis of food contaminants, allergens, and adulterants. These technologies combine novel biochemical recognition elements with state-of-the-art analytical platforms, addressing challenges posed by complex food matrices and processing-induced protein alterations.

Aptamer-based biosensors represent a prominent class of emerging tools. Aptamers are small, chemically synthesized oligonucleotides with high affinity and specificity for target proteins. Their robustness, ease of modification, and low batch variability make them ideal for developing portable and user-friendly detection devices. For example, an aptamer-based point-of-care diagnostic device has been successfully applied for detecting peanut allergens in diverse food matrices, achieving sensitivity down to 12.5 ppm and robustness across various food types [168]. Similarly, label-free aptamer-based colorimetric biosensors employing gold nanoparticles have been developed for rapid gliadin detection in gluten-containing foods, offering simple, cost-effective, and selective assays suitable for real-world samples [170]. These aptamer-based platforms enhance allergen detection accuracy, facilitating better consumer risk management.

Electrochemical biosensors have also gained traction due to their high sensitivity, rapid response, and potential for miniaturization. For instance, electrochemical aptasensors targeting *Staphylococcus aureus* surface proteins have been optimized using cyclic voltammetry to detect pathogens in food samples such as milk and apple juice with high specificity (Figure 7C) [171]. Additionally, immunosensors based on nanocomposites, such as silver nanoparticle-reduced graphene oxide combined with staphylococcal protein A, have been developed for sensitive detection of antibiotic residues like virginiamycin M1 in animal-derived foods, demonstrating excellent selectivity and reproducibility [172].

Mass spectrometry (MS)-based proteomics remains a cornerstone in detecting protein modifications related to food safety. Advances in MS techniques, including MALDI-TOF/TOF and LC–MS/MS, enable comprehensive profiling of allergens and processing-induced modifications. These methods provide detailed insights into protein structure, post-translational modifications, and the presence of chemical adducts, facilitating accurate allergen quantification and identification of adulterants [164,165]. Targeted MS approaches have proven superior to conventional ELISA kits (ioFront^®^ Soy ELISA Kit (LOQ: 1 ppm soy flour), Neogen Veratox^®^ Soy ELISA (LOQ: 2.5 ppm soy flour), and r-Biopharm RIDASCREEN^®^ FAST Soya ELISA (LOQ: 1 ppm soy protein)) in quantifying processed soy proteins, overcoming matrix effects and ingredient variability [166].

Nanotechnology integration enhances detection sensitivity and functionality of biosensors. Nanomaterials such as gold nanoparticles improve electrode conductivity and provide anchoring sites for biomolecules, enabling label-free electrochemical detection of gluten and other allergens [173]. Moreover, nanomaterials facilitate the development of nanosensors capable of detecting microbial contamination, hazardous chemicals, and pesticides, contributing to improved food safety monitoring (Figure 7D) [174].

Microfluidic paper-based analytical devices (μPADs) offer low-cost, rapid, and scalable alternatives to traditional lateral flow immunoassays for allergen detection. A single-piece lateral flow μPAD has been developed to detect ovalbumin in various food samples within 15 min, combining simplicity with sensitivity and potential for mass production [175].

Collectively, these emerging technologies leverage protein modifications as reliable biomarkers and employ innovative sensing strategies to overcome limitations of conventional detection methods. They contribute to enhancing food safety by providing tools that are sensitive, specific, rapid, and adaptable to diverse food matrices and processing conditions. Continued development and integration of these technologies hold promise for real-time, on-site food safety assessment, ultimately protecting public health and ensuring regulatory compliance.

#### 2.4.2. Applications in Environmental Monitoring

Protein modifications hold significant promise for environmental pollution monitoring due to their ability to serve as sensitive and specific biomarkers reflecting exposure to diverse environmental agents. Chemical modifications of proteins, including PTMs and adduct formations, can indicate the presence and intensity of pollutants in the environment. For example, human serum albumin (HSA) modifications at specific residues have been linked to ambient air pollutant exposure, revealing a complex landscape of protein adducts that correlate with toxicant levels [176]. Such modifications can be harnessed to develop biosensors that detect environmental contaminants with high specificity. Additionally, epitranscriptomic changes, which involve chemical modifications on RNA and their regulatory proteins, are influenced by environmental exposures and may contribute to disease development, suggesting that monitoring these modifications could provide early warning signals for environmental hazards [177]. The dynamic nature of protein and RNA modifications under environmental stress underscores their potential utility as biomarkers for real-time environmental monitoring. Furthermore, engineered biological systems, such as genetically modified marine bacteria with enhanced biofilm formation and biosensing capabilities, demonstrate how protein modifications can be exploited to create living sensors that detect environmental parameters like temperature and oxygen levels, integrating sensing with data storage via CRISPR-based systems (Figure 7E) [178]. These advances highlight the multifaceted potential of protein modifications in environmental monitoring, from molecular biomarkers to engineered biosensors, offering sensitive, specific, and adaptable tools for detecting and quantifying pollution.

Recent technological progress has significantly advanced the application of protein modifications in environmental monitoring, enhancing sensitivity, specificity, and robustness of biosensing platforms. Nanopore sequencing technology exemplifies a cutting-edge approach, enabling single-molecule analysis of protein modifications with high accuracy and real-time monitoring capabilities. This technique has been applied to detect protein post-translational modifications and unfolding, providing insights into molecular changes induced by environmental factors [179]. Complementing this, the development of efficient computational workflows for analyzing proteome-wide oxidative modifications, such as those generated by fast photochemical oxidation of proteins (FPOP), has facilitated large-scale structural and functional studies of proteins under environmental stress [180]. Surface modification strategies of nanomaterials, including graphene and graphene oxide, have enhanced biosensor performance by improving biomolecule immobilization and signal transduction, thereby increasing stability and sensitivity in complex environmental samples [181,182]. Moreover, innovative biosensing platforms employing functional tetrahedral DNA nanostructures (FTDN) have been developed for multiplexed detection of environmental biomarkers, demonstrating versatility in detecting nucleic acids, proteins, and metal ions relevant to pollution monitoring (Figure 7F) [183]. Advances in polymer coatings, such as liquid-like polydimethylsiloxane brushes, have improved electrode anti-biofouling properties, enabling stable and continuous monitoring of reactive oxygen species in bacteria-rich environments, which is critical for environmental sensing applications [184]. Additionally, novel universal biosensing platforms based on rapid direct native protein modification have been introduced, simplifying detection assays and enhancing sensitivity for environmental analytes [185]. Collectively, these technological innovations underscore a trend toward integrating protein modification detection with advanced materials, computational analysis, and engineered biological systems, paving the way for next-generation environmental monitoring tools that are highly sensitive, selective, and capable of real-time operation under diverse conditions.

### 2.5. Current Challenges and Future Directions

#### 2.5.1. Detection Sensitivity and Specificity

The sensitivity and specificity of protein modification-based biosensors are critical for their use in biological sensing and clinical diagnostics. Although significant progress has been made, current technologies still face inherent limitations that affect detection performance. For example, surface plasmon resonance (SPR) biosensors enable label-free and highly sensitive detection but often require further improvement to detect low-abundance viral proteins in complex samples. Modifying SPR sensors with nanomaterials like graphene oxide (GO) can enhance sensitivity by offering abundant functional groups for biomolecule immobilization and improving biocompatibility. However, achieving ultra-low detection limits and high specificity in heterogeneous matrices remains challenging [186]. Organic field-effect transistor (OFET)-based biosensors use organic semiconductor materials for signal amplification and label-free detection. Nevertheless, their performance is often affected by environmental factors and nonspecific binding, which reduces specificity and reproducibility [187]. Electrochemical biosensors are widely employed for protein detection due to their high sensitivity and operational simplicity. Yet, they are prone to biofouling caused by nonspecific adsorption of proteins and other biomolecules. This fouling gradually degrades sensor stability and specificity [76]. In semiconductor-based biosensors such as silicon nanowire field-effect transistors (SiNW-FETs), the Debye shielding effect considerably lowers sensitivity under physiological ionic strength. Overcoming this limitation often requires complex device designs or surface modifications [188].

Additionally, detecting post-translational modifications (PTMs) like ubiquitination and glycosylation remains difficult due to their low abundance and dynamic nature. Highly specific recognition elements and signal amplification strategies are essential but are not always available or sufficiently robust [189,190]. The utility of a PTM as a biomarker is context-dependent, dictated by the biological nature of the disease. For dynamic, pathway-driven diseases like cancer, phosphorylation is often the marker of choice due to its rapid reversibility and pivotal position in cellular signaling networks. Aberrant phosphorylation patterns directly mirror the dysregulation of growth and survival pathways, providing a mechanistic explanation for disease progression and a quantifiable metric for treatment efficacy. In contrast, for pathologies involving chronic inflammation or long-term tissue remodeling, more stable modifications such as advanced glycation end products (AGEs) may serve as better cumulative markers of disease burden [191]. for viral infections, how glycosylation is key because viruses co-opt host machinery to shield epitopes and mediate entry, making glycan profiles both a pathogen-specific signature and an indicator of immune evasion [192]. The complexity of biological samples, combined with the need for multiplexed detection of multiple protein modifications, further complicates achieving high sensitivity and specificity. Thus, current biosensor technologies face challenges including limited detection limits, nonspecific binding, matrix interference, and insufficient multiplexing capabilities, which collectively constrain their clinical and research utility.

To address these limitations, various strategies have been developed to enhance the sensitivity and specificity of protein modification biosensors. Nanomaterial-based sensor surface modifications have emerged as a powerful approach; for example, the integration of GO and MXenes with gold nanoparticles (AuNPs) significantly increases the active surface area and provides abundant binding sites, resulting in enhanced signal transduction and improved detection limits for viral proteins and exosome surface markers [108,185]. The use of peptide-guided assembly of silver nanoparticles (AgNPs) allows for signal amplification via in situ formation of conductive networks, enabling ultra-sensitive detection of cancer biomarkers such as HER2 with detection limits in the picogram per milliliter range [112]. Amplification techniques combining CRISPR/Cas systems with strand displacement amplification have been successfully employed to detect RNA modifications like N6-methyladenine (m6A) with femtomolar sensitivity, demonstrating the power of enzymatic cascade amplification for enhancing biosensor performance [118]. Antifouling surface engineering using zwitterionic peptides and engineered branching peptides improves biosensor specificity by minimizing nonspecific adsorption and biofouling in complex biological fluids, thereby maintaining sensor stability and accuracy [76,193]. Advanced device architectures, such as three-dimensional stacked silicon nanosheet FETs, overcome the Debye shielding effect by enabling probe molecule modification within stacked cavities, achieving ultra-high sensitivity in high ionic strength environments [188]. Label-free detection methods utilizing antimicrobial peptides as recognition elements combined with deep learning algorithms for impedance data analysis have demonstrated rapid and highly specific detection of viral proteins like HIV gp41 [194]. Furthermore, the development of covalent aptamer strategies stabilizes aptamer-target complexes, enhancing detection robustness and sensitivity even under harsh conditions, surpassing traditional antibody-based assays [195]. Integration of microfluidics with capacitive biosensors facilitates continuous and real-time monitoring with high sensitivity and specificity, as exemplified by C-reactive protein detection [196]. Lastly, multiplexed biosensor arrays constructed via in situ nanomaterial modification enable simultaneous detection of multiple cancer biomarkers with high throughput and accuracy, facilitating early disease diagnosis [197]. Collectively, these strategies leverage nanotechnology, enzymatic amplification, surface chemistry, device engineering, and computational analysis to overcome existing limitations, significantly advancing the sensitivity and specificity of protein modification biosensors for biomedical applications.

#### 2.5.2. Multiplexing and High-Throughput Strategies

To significantly enhance the multiplexing capability of protein modification detection, future platforms should explore integration with CRISPR-Cas systems. The programmability of CRISPR guide RNAs (gRNAs) can be harnessed to direct catalytically inactive Cas proteins (e.g., dCas9, dCas12) to specific genomic loci adjacent to genes encoding target proteins or to reporter constructs [198]. This spatial targeting enables the colocalization of multiple PTM-specific recognition elements (e.g., antibodies, nanobodies, or affimers), facilitating the simultaneous, spatially resolved detection of several PTMs on a single biosensor chip or in a microfluidic array. For instance, dCas proteins fused to different fluorescent proteins and targeted by unique gRNAs could provide a visual readout for concurrent monitoring of phosphorylation, acetylation, and methylation events. However, this promising approach is not without limitations [199]. Off-target effects remain a primary concern, as imperfect gRNA matching may lead to the mislocalization of detection complexes, compromising specificity and generating false-positive signals in a multiplexed setting. Additionally, the simultaneous operation of multiple CRISPR-Cas systems may lead to steric hindrance and signal crosstalk, while the large size of Cas protein fusions could impact the kinetics and sensitivity of PTM binding. Addressing these challenges will require the engineering of high-fidelity Cas variants, optimized delivery strategies for in-cell sensing, and sophisticated computational tools for gRNA design and signal deconvolution [200].

#### 2.5.3. Clinical Translation of Applications

The translation of protein modification-based biosensors and diagnostic tools from laboratory research to clinical application faces multiple significant barriers. Firstly, the complexity of biological samples in clinical settings presents a formidable challenge. Biosensors developed under controlled laboratory conditions often encounter interference from biofoulants such as proteins, cells, polysaccharides, and lipids present in blood, saliva, or other bodily fluids, which can severely affect sensor reliability and stability [76]. This necessitates the development of robust antifouling strategies and surface modifications to maintain sensor performance in complex biofluids. Secondly, reproducibility and standardization remain critical issues. Many biosensors utilize nanomaterials like graphene, gold nanoparticles, or MXenes to enhance sensitivity and selectivity [181,201,202], but variability in synthesis, functionalization, and immobilization techniques can lead to inconsistent sensor performance. This complicates regulatory approval and clinical adoption. Thirdly, the integration of biosensors into user-friendly, cost-effective, and scalable platforms suitable for point-of-care (POC) diagnostics is still under development. While advanced biosensors such as nanostructured mesoporous gold electrodes have demonstrated excellent sensitivity for detecting phosphorylated proteins relevant to cancer [38], their fabrication complexity and the need for specialized equipment limit widespread clinical use. Moreover, signal transduction mechanisms, including electrochemical, optical, and electrogenerated chemiluminescence methods, must be optimized for stability and minimal background noise in clinical environments [119,203]. Another barrier is the challenge of detecting PTMs with sufficient specificity and sensitivity. PTMs are often present in low abundance and can be transient or heterogeneous, complicating their reliable quantification. Mass spectrometry offers high accuracy but is costly and not conducive to rapid diagnostics [204]. Biosensor platforms must therefore balance sensitivity with operational simplicity. Furthermore, the Debye shielding effect in semiconductor biosensors can reduce detection sensitivity in physiological ionic strengths, necessitating innovative device architectures such as 3D stacked silicon nanosheet FETs to overcome this limitation [188]. Finally, clinical validation requires extensive testing with diverse patient samples to establish diagnostic accuracy, reproducibility, and clinical utility. Many biosensors remain at the proof-of-concept stage, lacking large-scale clinical trials and regulatory approval pathways. The complexity of protein modifications, the need for multiplexed detection of biomarkers, and integration with data analytics and AI for interpretation further complicate translation efforts [205]. In summary, the barriers to clinical translation include biological sample complexity, reproducibility and standardization challenges, fabrication and operational complexity, sensitivity and specificity for PTMs, and the need for rigorous clinical validation and regulatory compliance. Addressing these obstacles is essential for the successful deployment of protein modification-based biosensors in clinical diagnostics.

Future research aimed at overcoming the translational barriers of protein modification-based biosensors should focus on several key areas. One promising direction is the development of antifouling surface modifications and biocompatible nanomaterials that maintain sensor performance in complex biological fluids. For instance, peptide-functionalized titanium carbide MXenes have been engineered as biosensing interfaces to assay PTM enzyme activity with high sensitivity and selectivity, demonstrating potential for clinical diagnosis [202]. Similarly, hybrid nanocomposites combining graphene derivatives with conductive polymers have been designed to facilitate direct antibody immobilization without additional surface preparation, enhancing reproducibility and simplifying sensor fabrication [206]. Advances in nanofabrication techniques, such as wafer-level processes for gallium arsenide high-electron-mobility transistor biosensors, enable ultra-sensitive and rapid detection of viral proteins without complex nanomaterial modifications, pointing toward scalable clinical platforms [207]. Another critical area is the integration of high-throughput and multiplexed detection capabilities. Platforms like Sensor-Integrated Proteome On Chip (SPOC^®^) allow simultaneous capture and real-time kinetic analysis of thousands of folded proteins on a single biosensor chip, facilitating comprehensive biomarker profiling for clinical applications [208]. This approach can enhance diagnostic accuracy by enabling multi-analyte detection. Overcoming physical limitations such as the Debye shielding effect is also essential. Innovative device architectures, including three-dimensional stacked silicon nanosheet FET biosensors, have demonstrated enhanced sensitivity at physiological ionic strengths, enabling point-of-care testing for nucleic acids and potentially proteins [188]. The incorporation of machine learning and advanced signal processing algorithms can improve the resolution and accuracy of biosensors, particularly in nanopore-based protein sensing, which holds promise for detecting amino acid sequences and PTMs with label-free, single-molecule sensitivity [209]. Furthermore, the development of modification-free biosensing strategies, such as the in situ formation of silver nanoparticle networks for signal amplification, offers simplified procedures and potential for regeneration and reuse, which are advantageous for clinical deployment [210]. Clinical translation also requires rigorous validation with patient-derived samples and standardization of protocols. The establishment of comprehensive databases and cloud computing resources for proteomic data, including PTMs and sequence variants, can facilitate biomarker discovery and validation [211]. Additionally, the design of biosensors compatible with point-of-care settings, including portable, low-cost, and user-friendly devices such as paper-based lateral flow immunoassays enhanced by AI-driven analysis, can accelerate clinical adoption [205]. Finally, interdisciplinary collaboration among materials scientists, bioengineers, clinicians, and regulatory experts is crucial to address the multifaceted challenges in sensor development, clinical validation, and regulatory approval. In conclusion, future research should focus on developing robust, reproducible, multiplexed, and user-friendly biosensing platforms, leveraging advanced nanomaterials, innovative device architectures, and computational tools to enable the successful clinical translation of protein modification-based diagnostics. Table 2 succinctly compares the key regulatory hurdles and considerations for PTM-based biosensors against those for traditional in vitro diagnostics (IVDs), specifically highlighting challenges related to analytical validation, clinical validation, manufacturing, and quality control under frameworks like those of the FDA and EMA.

## 3. Conclusions

Protein modifications, particularly PTMs such as phosphorylation, glycosylation, and methylation, play critical roles in regulating protein structure, function, and interactions, thereby influencing numerous biological processes and disease states. The ability to detect and analyze these modifications has become essential in biosensing and diagnostic applications, as they often serve as biomarkers for various diseases including cancer, neurodegenerative disorders, and infectious diseases. Advances in biosensor technologies have leveraged the specificity and sensitivity of protein modifications to achieve precise and rapid detection. For instance, phosphoproteomics, which focuses on identifying phosphorylated proteins, benefits from biosensors that convert biological interactions into measurable signals such as electrochemical, optical, or fluorescence outputs, enabling the detection of aberrant phosphorylation patterns linked to disease [212]. Similarly, glycosylation, a key modification affecting protein stability and therapeutic efficacy, is increasingly analyzed using electrochemical biosensors that provide rapid, sensitive, and cost-effective glycan profiling critical for biotherapeutic quality control [65]. The integration of nanomaterials such as graphene, gold nanoparticles, and MXenes with biosensors enhances biomolecule immobilization and signal transduction, improving detection sensitivity and stability [181,213,214]. Moreover, innovative biosensors employing FRET, magnetoresistance, and SPR techniques have been developed to monitor dynamic protein modifications and interactions in real time, facilitating early diagnosis and monitoring of diseases like Alzheimer’s and cancer [203,215]. The modification of biosensor surfaces with antifouling coatings and functional peptides further enhances specificity and reduces nonspecific binding, which is crucial for reliable detection in complex biological samples [76,216]. Additionally, the engineering of proteins with site-specific incorporation of modified amino acids, such as L-DOPA, expands the toolkit for designing biosensors with tailored recognition and signaling capabilities [217]. Collectively, protein modifications underpin the development of highly sensitive, selective, and versatile biosensors that transform molecular interactions into quantifiable signals, thereby advancing the fields of biomedical diagnostics, environmental monitoring, and therapeutic development. The ongoing challenges include improving multiplexing capabilities, real-time in vivo monitoring, and integration with wearable and implantable devices, which are being addressed through novel material modifications and biosensor architectures [205,218]. Thus, protein modification is fundamental to the innovation and efficacy of biosensing and diagnostic platforms, offering profound implications for precision medicine and public health.

The future trajectory of protein modification in biosensing and diagnostics underscores a critical need for continued research to overcome current limitations and fully realize its application potential. Despite significant advances in chemical, enzymatic, and physical modification techniques, challenges such as precise control over modification sites, stability of modified proteins, and biocompatibility remain. For instance, site-specific protein modifications, particularly at the N-terminus, have demonstrated transformative potential in therapeutic protein engineering and functional biomaterials, yet integrating artificial intelligence with bioorthogonal chemistry is proposed as a future direction to expand the modification toolbox and explore novel applications in drug development and basic research [219]. Similarly, chemical modifications like succinylation have improved protein functionalities such as solubility and emulsification, but issues such as controlling modification degree and ensuring long-term chemical stability require further investigation to facilitate industrial acceptance [220]. In the realm of biosensor development, surface modification strategies that enhance antifouling properties and protein immobilization efficiency are essential for improving sensitivity and specificity. The grafting of zwitterionic peptides onto hyaluronic acid-modified surfaces exemplifies such innovation, offering enhanced protein resistance and suggesting new avenues for biosensor surface engineering [216]. Moreover, the integration of nanomaterials and peptide-guided assemblies has shown promise in creating highly sensitive and specific biosensors for cancer biomarkers, yet further research is needed to optimize these systems for clinical applications [112]. The application of advanced protein modifications extends beyond food and biomedical sectors into gene therapy, where chemical modifications of viral capsids like adeno-associated virus (AAV) vectors are being explored to improve targeting and reduce immune responses, though challenges related to protein stoichiometry and vector fitness persist [221,222]. Emerging post-translational modifications such as lysine succinylation and lactylation reveal new regulatory mechanisms in diseases including cancer and neurological disorders, highlighting the importance of elucidating modification-specific biological functions and developing targeted therapeutics [223,224,225]. Furthermore, the advancement of biosensors capable of multiplexed detection addresses tumor heterogeneity and improves diagnostic precision yet demands further refinement in biomarker selection and sensor design [111]. The convergence of synthetic biology, nanotechnology, and chemical biology is anticipated to drive the next generation of protein modifications and biosensors, enabling highly specific, stable, and multifunctional platforms for diagnostics and therapeutics. Future research should focus on integrating computational tools, optimizing modification chemistries, and conducting comprehensive safety evaluations to translate these innovations into clinical and industrial settings. Collectively, these efforts will unlock the full potential of protein modifications in biosensing and diagnosis, fostering breakthroughs in precision medicine, environmental monitoring, and food safety.

The continuous advancement of protein modification detection technologies is pivotal for expanding our understanding of protein functions and their roles in health and disease. Current methodologies encompass a broad spectrum, including immunological assays with modification-specific antibodies, mass spectrometry (MS)-based proteomics, affinity-based enrichment techniques, and emerging single-molecule sensing platforms. For instance, ubiquitination detection has evolved from antibody-based immunodetection to high-throughput approaches such as tandem hybrid ubiquitin-binding domain (ThUBD)-coated multi-well plates, which offer universal, sensitive, and specific detection of diverse polyubiquitin chain modifications across various biological samples [169]. Similarly, reverse phase protein array (RPPA) technology provides a robust antibody-based platform capable of profiling multiple PTMs like phosphorylation, methylation, and acetylation in a high-throughput manner, facilitating comprehensive proteomic analyses [226].

The integration of computational tools has become increasingly important to interpret complex PTM datasets. Novel algorithms such as RoLiM enable robust, unbiased deconvolution of linear motifs associated with diverse protein modifications at the proteome scale, addressing limitations of previous motif detection methods and enhancing the identification of substrate motifs relevant to kinase signaling pathways [227]. Mass spectrometry remains a cornerstone for detecting both enzymatic and nonenzymatic PTMs, with innovations like isotope dilution gas chromatography-mass spectrometry (SIM-GC/MS) enabling sensitive quantification of protein damage markers in clinical samples such as cerebrospinal fluid, thereby expanding the scope of PTM analysis beyond traditional proteomics [228].

Emerging chemical biology techniques, such as the incorporation of non-canonical amino acids (ncAAs), allow site-specific protein modifications that facilitate the detection and enrichment of modified proteins, exemplified by engineered SUMO-trapping bioconjugates for diagnostic applications [229]. Genetic code expansion technologies further enable co-translational incorporation of modified residues, offering precise control over PTMs for functional studies and potential therapeutic interventions [230]. These approaches underscore a trend towards more precise, site-specific, and functional interrogation of protein modifications.

Nanopore technology represents a transformative advancement in protein detection, offering label-free, single-molecule sensitivity with rapid detection capabilities. Both biological and solid-state nanopores have been adapted to detect protein sequences, structural variants, and PTMs, with ongoing developments in translocation control and signal processing enhancing their applicability in clinical diagnostics and biomarker discovery [219,231]. The potential for real-time, high-throughput, and miniaturized platforms positions nanopore sensing as a promising frontier for next-generation protein modification analysis.

Furthermore, the application of click chemistry has facilitated efficient and selective labeling of PTMs, such as palmitoylation and carbonylation, enabling detailed characterization of modification dynamics during pathological states like viral infection [232]. The integration of aptamer-based biosensors and DNA nanotechnology has also expanded the toolkit for sensitive and specific detection of protein modifications and related biomarkers, with potential for point-of-care diagnostics [163,233].

Despite these advances, challenges remain in achieving comprehensive, multiplexed, and quantitative detection of the vast array of protein modifications in diverse biological contexts. The complexity and dynamic nature of PTMs necessitate continuous innovation in detection sensitivity, specificity, throughput, and data analysis. Future directions include the development of integrated platforms combining chemical, biological, and computational methods to enable holistic profiling of protein modifications. The convergence of high-resolution mass spectrometry, single-molecule sensing technologies, and machine learning algorithms promises to unravel the functional landscape of protein modifications with unprecedented detail. Developing standardized, multiplexed electrochemical arrays capable of profiling a panel of 10 phospho-protein biomarkers directly from a finger-prick blood sample within 20 min is a key near-term goal [234].

In summary, the outlook for protein modification detection technologies is highly promising, driven by multidisciplinary innovations that enhance sensitivity, specificity, throughput, and functional insight. These advancements will not only deepen our understanding of proteome complexity but also accelerate the translation of protein modification knowledge into clinical diagnostics, therapeutic monitoring, and personalized medicine.

In conclusion, protein modifications represent a transformative frontier in the field of biosensing and diagnostics, offering unparalleled opportunities for early disease detection, food safety assurance, and environmental monitoring. From an expert perspective, the development of protein modification-based detection technologies has significantly expanded our capability to interrogate biological systems with high specificity and sensitivity, thereby enabling more precise and timely diagnostic interventions. The impact of protein modifications in biosensing is multifaceted. On one hand, these modifications serve as critical biomarkers that reflect dynamic physiological and pathological states, providing a rich source of diagnostic information beyond what traditional nucleic acid or metabolite-based assays can offer. On the other hand, the chemical versatility of protein modifications allows for the design of innovative sensor platforms that can selectively recognize and quantify target analytes in complex biological matrices. This dual advantage underscores the potential of protein modification-based biosensors to revolutionize clinical diagnostics by facilitating earlier and more accurate disease detection, which is crucial for improving patient outcomes. However, balancing the promise of these technologies with the current challenges is essential for their successful translation into clinical and real-world applications. Sensitivity and specificity remain primary concerns, as the detection of low-abundance protein modifications often requires sophisticated amplification strategies and highly selective recognition elements. Additionally, the heterogeneity of protein modifications and their dynamic nature pose significant analytical challenges, necessitating the development of robust, reproducible, and high-throughput detection methods. From a translational standpoint, integrating these biosensors into user-friendly platforms that meet regulatory standards and demonstrate clinical utility is critical for widespread adoption. To address these challenges, ongoing research must adopt a multidisciplinary approach that combines advances in chemistry, molecular biology, engineering, and data analytics. Innovations such as nanomaterial-enhanced sensors, multiplexed detection systems, and machine learning algorithms for signal interpretation are promising avenues that can enhance the performance and applicability of protein modification-based biosensors. Furthermore, fostering collaborations between academic researchers, clinicians, and industry stakeholders will accelerate the optimization and validation of these technologies, ensuring that they meet the practical demands of clinical diagnostics and environmental monitoring. In summary, while the field of protein modification-based biosensing is still evolving, its potential to transform biomedical diagnostics is undeniable. By carefully balancing the diverse research perspectives—ranging from fundamental biochemical insights to engineering innovations and clinical requirements—future developments are poised to overcome current limitations. This will ultimately lead to more sensitive, specific, and accessible diagnostic tools that can significantly impact public health, food safety, and environmental protection. As technological advancements continue to emerge, protein modification detection is expected to become an indispensable component of the next generation of biosensors, driving forward the precision medicine era and beyond.

## Figures and Tables

**Table 1:** The biological sensor platform for detecting cancer markers is used for the early diagnosis of cancer.

Biosensor Platforms	Target Protein	Sensitivity	Specificity	Reference
Electrokinetic principle-based biosensor	sEV surface membrane proteins	EGFR: 10% CD63: 3%	Specific interaction-based targeted detection	[105]
SERS biosensor (Q-structured TiO_x_ template, quantum-scale regulation + oxygen vacancy introduction)	EGFR	Detection limit as low as 1 nM; maximum enhancement factor (EF) of 3.4 × 10^7^	Demonstrated for breast and cervical cancer cell lines	[106]
Electrochemical biosensor based on peptide-guided assembly of silver nanoparticles (AgNPs)	Human epidermal growth factor receptor 2 (HER2)	Limit of detection (LOD) as low as 0.05 pg/mL	Distinguishes HER2^+^ and HER2^−^ breast cancer patients; applicable for serum HER2 detection with antifouling performance	[108]
Carbon dot-functionalized extended gate organic field effect transistor (OFET)	Carcinoembryonic antigen (CEA)	Limit of detection (LOD) as low as 2.7 pg/mL	High selectivity for carcinoembryonic antigen (CEA)	[110]
Electrochemical homogeneous bioplatform based on metal ions/SiO_2_NPs/magnetic beads	Estrogen receptor (ER), Progesterone receptor (PR), Human epidermal growth factor receptor 2 (HER2), Ki67	Linear range: 0–1000 pg/mL Detection limits: ER (1.8 pg/mL), PR (1.33 pg/mL), HER2 (2 pg/mL), Ki67 (10.36 pg/mL) Detection time: 140 min	Selectively detects the four target biomarkers; enables simultaneous diagnosis of 10 types of breast cancer directly in human serum	[111]

**Table 2:** Key Regulatory Hurdles: A Comparison of Traditional IVDs vs. PTM-Based Biosensors.

Regulatory Aspect	Traditional IVDs (e.g., Glucose, Cardiac Troponin)	PTM-Based Biosensors	Challenges & Considerations for PTM Biosensors
Analytical Validation	Well-established protocols for specificity, accuracy, precision, and linearity against a known analyte.	Must demonstrate high specificity for the PTM itself, distinct from the unmodified protein and other similar modifications.	Lack of universal gold-standard methods and certified reference materials for many PTMs complicates validation. Specificity assays must rule out cross-reactivity.
Clinical Validation	Clear clinical decision points and thresholds are often defined (e.g., diagnostic cutoff for myocardial infarction).	Must establish a robust correlation between the measured PTM level and a specific clinical status or outcome.	The dynamic and heterogeneous nature of PTMs makes defining clinically relevant thresholds complex. Requires large, well-characterized patient cohorts.
Standardization & Reproducibility	Focuses on reagent lot consistency and assay reproducibility across instruments and sites.	Must control for variability in nanomaterial synthesis, surface functionalization, and bioreceptor immobilization.	Reproducibility is a major hurdle due to complex fabrication. stringent control over manufacturing processes (CMC *) is critical for regulatory approval.
Stability & Shelf-Life	Testing focuses on reagent and signal stability under defined storage conditions.	Must ensure the stability of the delicate sensing interface (e.g., immobilized enzymes, antibodies, nanomaterials).	PTM sensitivity can degrade due to surface fouling or decomposition of biological recognition elements over time.
Quality Control	QC testing monitors performance against predefined specifications using control materials.	Requires development of novel control materials that faithfully represent the specific PTM target.	Creating stable, multiplexed control samples containing defined levels of specific PTMs is technically challenging and costly.

* CMC: Chemistry, Manufacturing, and Controls.

## Data Availability

Not applicable.

## Abbreviations

The following abbreviations are used in this manuscript:

PTMs	post-translational modifications
SPR	surface plasmon resonance
SERS	surface-enhanced Raman scattering
MAPK	mitogen-activated protein kinase
FRET	fluorescence resonance energy transfer
AD	Alzheimer’s disease
p-tau	phosphorylated tau proteins
acetyl-CoA	acetyl-Coenzyme A
FET	field-effect transistors
QCM	quartz crystal microbalance
AFP	alpha-fetoprotein
PSA	prostate-specific antigen
CV	cyclic voltammetry
DPV	differential pulse voltammetry
EIS	electrochemical impedance spectroscopy
PEG	polyethylene glycol
MIP	Molecularly imprinted polymers
MAA	milk amyloid A
HSP70	heat shock protein 70
CTGF	connective tissue growth factor
HE4	human epididymis protein 4
MOF	microstructured optical fiber
LPFG	long-period fiber gratings
TFBG	tilted fiber Bragg gratings
FOEW	fiber-optic evanescent wave
BODIPY	boron dipyrromethene
FLRDS	Fiber loop ringdown spectroscopy
VG	voltage
Cq	capacitance of graphene
SPE	screen-printed electrodes
OFET	Organic field-effect transistors
CEA	carcinoembryonic antigen
IBV	infectious bronchitis virus
HSV-1	Herpes simplex virus 1
FA	formaldehyde
PMIF	protein modifications induced by formaldehyde
BITC	benzyl isothiocyanate
MS	Mass spectrometry
μPADs	Microfluidic paper-based analytical devices
HSA	human serum albumin
FPOP	fast photochemical oxidation of proteins
FTDN	functional tetrahedral DNA nanostructures
GO	graphene oxide
OFET	organic field-effect transistor
SiNW-FETs	silicon nanowire field-effect transistors
AuNPs	gold nanoparticles
AgNPs	silver nanoparticles
POC	point-of-care
AAV	adeno-associated virus
ThUBD	tandem hybrid ubiquitin-binding domain
RPPA	reverse phase protein array
